# Loss of X-linked *Protocadherin-19* differentially affects the behavior of heterozygous female and hemizygous male mice

**DOI:** 10.1038/s41598-017-06374-x

**Published:** 2017-07-19

**Authors:** Shuichi Hayashi, Yoko Inoue, Satoko Hattori, Mari Kaneko, Go Shioi, Tsuyoshi Miyakawa, Masatoshi Takeichi

**Affiliations:** 1 Laboratory for Cell Adhesion and Tissue Patterning, RIKEN Center for Developmental Biology, 2-2-3 Minatojima-Minamimachi, Chuo-ku, Kobe 650-0047 Japan; 2 Division of Systems Medical Science, Institute for Comprehensive Medical Science, Fujita Health University, 1-98 Dengakugakubo, Kutsukake-cho, Toyoake 470-1192 Japan; 3 Animal Resource Development Unit, RIKEN Center for Life Science Technologies, Kobe, 650-0047 Japan; 4 Genetic Engineering Team, RIKEN Center for Life Science Technologies, Kobe, 650-0047 Japan; 5 Section of Behavior Patterns, Center for Genetic Analysis of Behavior, National Institute for Physiological Sciences, 38 Nishigonaka, Okazaki, Aichi 444-8787 Japan; 6 Present Address: Department of Physiology, Anatomy and Genetics, Le Gros Clark Building, University of Oxford, South Parks Road, Oxford, OX1 3QX UK

## Abstract

Mutations in the X-linked gene *Protocadherin-19* (*Pcdh19*) cause female-limited epilepsy and mental retardation in humans. Although Pcdh19 is known to be a homophilic cell-cell adhesion molecule, how its mutations bring about female-specific disorders remains elusive. Here, we report the effects of *Pcdh19* knockout in mice on their development and behavior. *Pcdh19* was expressed in various brain regions including the cerebral cortex and hippocampus. Although Pcdh19-positive cells were evenly distributed in layer V of wild-type cortices, their distribution became a mosaic in *Pcdh19* heterozygous female cortices. In cortical and hippocampal neurons, Pcdh19 was localized along their dendrites, showing occasional accumulation on synapses. *Pcdh19* mutants, however, displayed no detectable abnormalities in dendrite and spine morphology of layer V neurons. Nevertheless, *Pcdh19* hemizygous males and heterozygous females showed impaired behaviors including activity defects under stress conditions. Notably, only heterozygous females exhibited decreased fear responses. In addition, Pcdh19 overexpression in wild-type cortices led to ectopic clustering of Pcdh19-positive neurons. These results suggest that Pcdh19 is required for behavioral control in mice, but its genetic loss differentially affects the male and female behavior, as seen in human, and they also support the hypothesis that the mosaic expression of *Pcdh19* in brains perturbs neuronal interactions.

## Introduction

Impairments in neural development are implicated in brain disorders such as autism and schizophrenia^1^. Although genetic factors are thought to be important in many such disorders, the underlying molecular mechanisms remain elusive in most cases. Establishing knockout mouse models for human disorders and analyzing their phenotypes are important steps for understanding the physiological functions of responsible genes as well as the pathogenesis of disorders caused by their mutations.

Protocadherins (Pcdhs) are a group of cadherin superfamily proteins, which consist of two subgroups—clustered and non-clustered Pcdhs^2^—and each subgroup is further divided into subfamilies. In general, they localize in the cell membranes and undergo homophilic interactions at the apposed cell surfaces, regulating cell-cell contacts^3^. Both subgroups are expressed in the central and peripheral nervous systems during development as well as at adult stages. Although their physiological functions have not been fully elucidated, recent studies suggest that clustered Pcdhs regulate inter-neuronal recognition, based on their subtype-specific binding properties^3–5^. Non-clustered Pcdhs are divided into a few subfamilies, and the δ2-protocadherin subfamily consists of several members including Pcdh10, 17 and 19. Previous studies of δ2-protocadherins suggested that they are involved in development of the central nervous system: Pcdh19 in tectum formation in zebrafish^6^, Pcdh17 in axon extension in mice^7^, and Pcdh8/Arcadlin and Pcdh17 in synapse formation and/or plasticity in mice^8, 9^.

In humans, mutations in the *PCDH19* gene, which is located on the X chromosome, have been identified as a cause of epilepsy-intellectual disability in females (EFMR)^10^. The inheritance pattern of this disorder is unusual in familial cases: only females with heterozygous *PCDH19* gene mutations are affected, whereas males with hemizygous mutations are not. The majority of sporadic cases have also been reported in females^11^, although some reports showed rare cases of male patients who have a *PCDH19* mutation or deletion with a mosaicism^12–14^. Despite increasing clinical evidence that mutations in the *PCDH19* gene cause EFMR^11^, it still remains largely unclear how *PCDH19* regulates the development and functions of the nervous system and how its mutations cause human disease.

In this study, we performed phenotypic analysis of *Pcdh19* knockout mice. Expression of *Pcdh19* in the cerebral cortex started in embryonic stages and persisted into adulthood. Its expression includes layer V of the cortex and CA1 of hippocampus. *Pcdh19* expression in heterozygous female mutants became patchy at the cortex, and was not symmetrical between the left and right hemispheres. Although there were no detectable abnormalities in synapse formation in cortical layer V neurons, we found that *Pcdh19* heterozygous female and hemizygous male mutants showed impaired activity in some behavioral tests. Notably, phenotypes were not identical between the heterozygous female and hemizygous male mutants, and some phenotypes were only prominent in females. Our results suggest that behaviors seen in *Pcdh19* mouse mutants at least in part mimic female-specific brain disorders observed in human EFMR. The present findings also support the hypothesis of cellular interference to explain the pathophysiology of EFMR.

## Results

### Pcdh19 is expressed in cortical layer V and hippocampal CA1 in mouse brains

**Figure 1:**
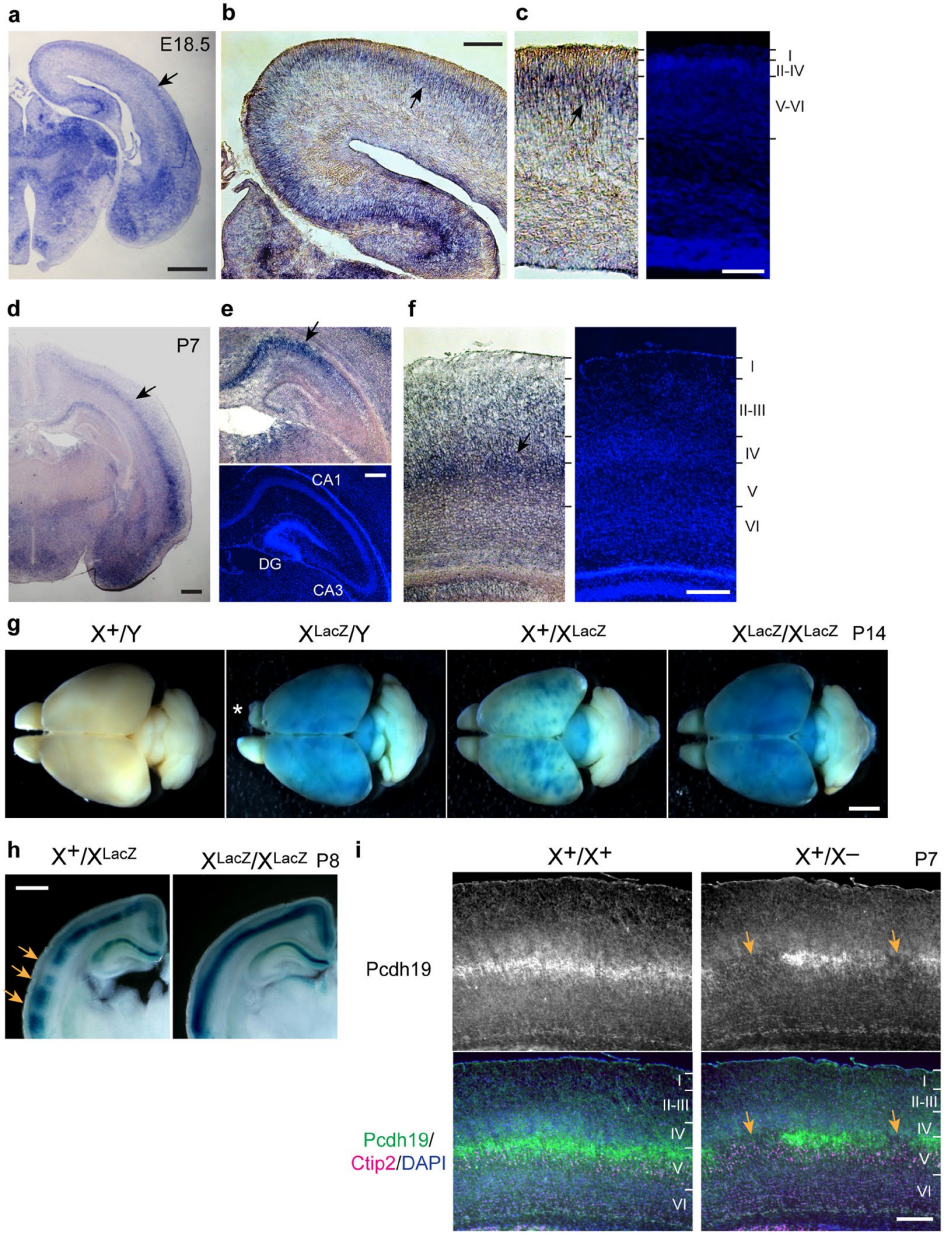
*Pcdh19* expression in the cerebral cortex and hippocampus becomes patchy in heterozygous female mutants. (**a**–**c**) *In situ* hybridization of *Pcdh19* mRNA in E18.5 brain. Signals were detected in the cerebral cortex and thalamus (**a**). *Pcdh19* mRNA showed a continuous distribution in the cortex and hippocampal CA1 region (**b**). *Pcdh19* mRNA localized mainly in the upper part of the deep layer (layer V) (**c**). (**d**–**f**) *In situ* hybridization of *Pcdh19* mRNA in P7 brain. *Pcdh19* expression was detected in the cortex and hippocampal region (**d**). Higher magnification images show that *Pcdh19* localized in the hippocampal CA1 (**e**) and cortical layer V (**f**). (**g**) X-gal staining of whole brains from P14 mice to detect the activity of *LacZ* that was knocked-in to the *Pcdh19* gene locus. Note that the distribution of X-gal signals becomes patchy in *Pcdh19* heterozygous female mutants (X^+^X^LacZ^), whereas the signals were evenly detected in the whole cortical region in hemizygous male (X^LacZ^Y) and homozygous female mutants (X^LacZ^X^LacZ^). Wild-type male (X^+^Y) brain was used as a negative control. Asterisk in the image of X^LacZ^Y indicates dissection artifacts. (**h**) Coronal slices of brain from heterozygous (X^+^X^LacZ^) and homozygous (X^LacZ^X^LacZ^) female mice at P8 after X-gal staining. Arrows indicate X-gal-negative regions in the heterozygous mutant brain. (**i**) Localization of Pcdh19 (green), Ctip2 (magenta) and DAPI (blue) in layer Va in the somatosensory cortex of wild-type (X^+^X^+^) and *Pcdh19* heterozygous (X^+^X^−^) female mice at P7. Arrows indicate a Pcdh19-negative region in the heterozygous mutant brain. We used 14-μm-thick (**a**–**f**) and 10-μm-thick (**i**) sections. Scale bars, 2 mm in (**g**) 1 mm in (**a**,**d**,**h**) 200 μm in (**b**,**c**,**e**,**f**) 100 μm in (**i**).

Previous studies reported that *Pcdh19* is expressed in developing mouse brains at embryonic and postnatal stages^15, 16^. We first confirmed the brain expression of *Pcdh19* using E18.5 and P7 mice. *Pcdh19* mRNA was distributed in the cerebral cortex and in some of the thalamic nuclei at E18.5 (Fig. 1a). In the cortex, *Pcdh19* expression was detected in layer V, and this expression was extended to the hippocampal CA1 region (Fig. 1b,c). The *Pcdh19* expression in cortical layer V and hippocampal CA1 persisted in P7 brains (Fig. 1d–f).

To examine the expression of Pcdh19 proteins, we generated an antibody specific to Pcdh19, and this antibody recognized a ∼150 kDa protein in lysates from wild-type male (X^+^/Y) and female mice (X^+^/X^+^) (Supplementary Fig. S1a). In *Pcdh19* heterozygous (X^+^/X^−^) and null mutants (X^−^/Y, X^−^/X^−^), the details of which are described in the following paragraphs, the ∼150 kD signal decreased and disappeared, respectively. These results suggest that this antibody specifically recognizes the Pcdh19 protein. There was no detectable difference in the amount of Pcdh19 protein between male and female wild-type mice at P7. We then examined the expression level of Pcdh19 proteins using lysates of the cerebral cortex and hippocampus from different stages of developing and adult mouse brains (Supplementary Fig. S1b). Expression of Pcdh19 proteins was detected at E10.5 and later developmental stages, continuing in the adult cortex and hippocampus. Immunostaining analysis confirmed that Pcdh19 localizes mainly to layer V in the cerebral cortex, as found for its mRNA. More detailed analysis showed that Pcdh19 localized in layer Va in the somatosensory cortex of postnatal brains, where its localization was nearly complementary to that of Ctip2, a layer Vb marker (Supplementary Fig. S1c).

To explore the function of *Pcdh19*, we generated *Pcdh19* knockout mice (Supplementary Fig. S2a,b). X-gal staining of postnatal brains from different genotypes of *Pcdh19* mutants, in which *LacZ* was knocked into the *Pcdh19* gene locus, showed that the *LacZ* staining signals were widely distributed over the cortex in hemizygous male (X^LacZ^Y) and homozygous female (X^LacZ^/X^LacZ^) mutants (Fig. 1g). By contrast, heterozygous females (X^+^X^LacZ^) showed a patchy distribution of *LacZ* signals (Fig. 1g). The patchy signals were randomly distributed, resulting in their distinct distributing pattern between the right and left hemispheres in the heterozygous mutants. Cross sections of brains confirmed that *LacZ*-positive regions were discontinuous in heterozygous females (Fig. 1h, arrows in the left panel), whereas these were continuous in homozygous females throughout the cortex (Fig. 1h, right panel). Distribution of Pcdh19 proteins also became patchy in layer Va of the somatosensory cortex of heterozygous females (Fig. 1i, arrows in the right panel). In young adult (P35) heterozygous brains, *LacZ* signals again showed a patchy distribution in layer V (Supplementary Fig. S2c), suggesting that the mosaic expression of *Pcdh19* in the cortex persists in adult brains. Previous studies indicate that sister neurons from the same clone show a columnar distribution in the cortex^17^, and the distribution of X-linked genes differs between the right and left hemispheres even in wild-type brains^18^. Thus, it is most likely that the patchy and asymmetrical expression of Pcdh19 was caused by random inactivation of an X chromosome bearing the *Pcdh19* gene, which results in a mosaic distribution of *Pcdh19*-positive and -negative neurons in heterozygous female cortices.

Previous studies reported that *Pcdh19* expression also occurs in layers II/III in addition to layer V in mature brains^19^. Our X-gal staining only faintly detected *LacZ*-positive cells in layers II/III at the first and second postnatal weeks (Supplementary Fig. S2d,e), suggesting that *Pcdh19* expression may progressively increases in layers II/III during postnatal development. Despite the complete or mosaic loss of *Pcdh19* expression in the mutants, their somatosensory cortex showed apparently normal layer organization (Supplementary Fig. S2f).

### Overexpression of Pcdh19 induces clustering of neurons in the cerebral cortex

**Figure 2:**
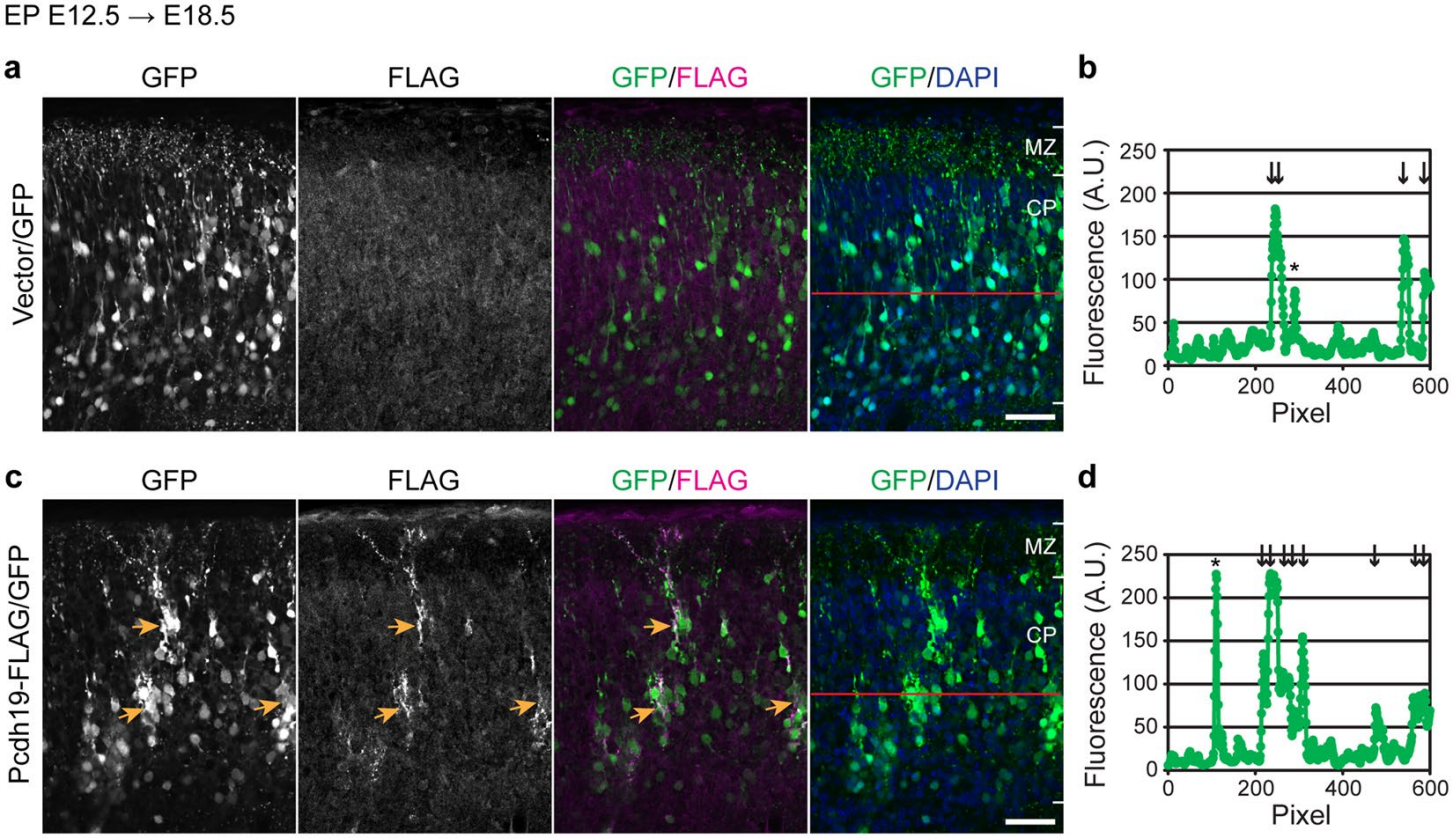
Overexpression of Pcdh19 induces clustering of neurons in the cerebral cortex. (**a**–**d**) pNeuroD-Cre driven expression of GFP without (**a**,**b**) or with Pcdh19b-FLAG (**c**,**d**) in cortical neurons. DNA transfer was performed by *in utero* electroporation at E12.5. (**a**) GFP-labeled neurons were distributed in the cortical plate. (**b**) Fluorescence intensities along the red line indicated in the rightmost image in **a**. Arrows indicate GFP-positive cell bodies, and asterisk indicates a neuronal process along the line. (**c**) GFP-labeled neurons expressing Pcdh19-FLAG formed clusters in the cortical plate. (**d**) Fluorescence intensities along the red line indicated in the rightmost image in (**c**). Arrows indicate the GFP-positive cell bodies, and asterisk indicates a neuronal process along the line. Scale bar, 50 μm.

Although X-chromosome inactivation explains the mosaic distribution of *Pcdh19*-positive neurons, we tested the possibility that the homophilic binding property of protocadherins may also contribute to the patchy distribution of these neurons. We exogenously expressed Pcdh19 in post-mitotic neurons of wild-type cortices by *in utero* electroporation of E12.5 brains with a vector that encodes pNeuroD-Cre, which expresses Cre recombinase under the neuron-specific promotor NeuroD, and two conditional vectors that encode GFP and Pcdh19-FLAG in a Cre-dependent manner. In control brains that express only GFP, labelled neurons were randomly distributed in the cortical plate (Fig. 2a,b). In brains expressing GFP and Pcdh19-FLAG, on the other hand, labeled neurons formed clusters, although they normally migrated into the cortical plate (Fig. 2c,d, arrows). In the clustered neurons, exogenous Pcdh19 proteins accumulated between their interfaces (Fig. 2c, FLAG). This result suggests that a local increase of Pcdh19 forces a group of cells to stay together via its homophilic binding property.

### Pcdh19 is localized along dendrites including spines

**Figure 3:**
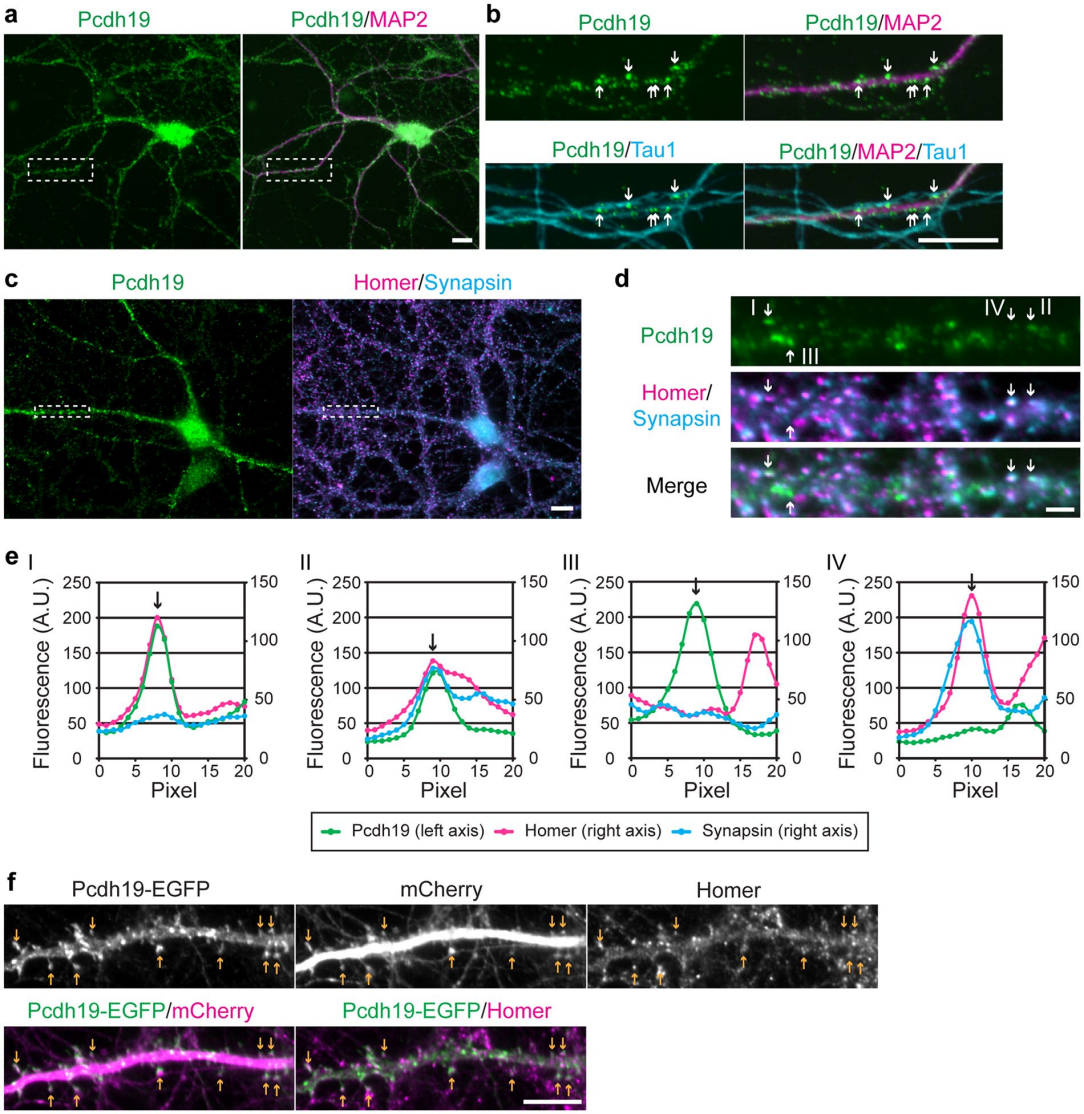
Distribution of Pcdh19 along dendrites of hippocampal neurons. (**a**,**b**) Pcdh19 protein localization along neurites of cultured hippocampal neurons at DIV7. Anti-MAP2 and Tau1 antibodies were used to visualize dendrites and axons, respectively. (**a**) Pcdh19 localization along dendrites. Tau1 signals are not shown. (**b**) Enlarged images of the boxed region in (**a**). Pcdh19 localizes along dendrites and their signals overlapped with axons along the dendrite. (**c**) Pcdh19 protein localization along dendrites of hippocampal neurons at DIV21. Anti-Homer-1 (Homer) and Synapsin-1/2 (Synapsin) antibodies were used to detect post- and pre-synapses, respectively. (**d**) Localization of Pcdh19, Homer-1 and Synapin-1/2 along the dendrite indicated in the box in (**c**). Representative Pcdh19 signals are indicated by arrows with labels that correspond to those in (**e**). (**e**) Fluorescence intensities at the points indicated by arrows in (**d**). I, colocalization of Pcdh19 with Homer-1; II, colocalization of Pcdh19 with both Homer-1 and Synaptin-1/2; III, no colocalization of Pcdh19 with Homer-1 or Synapsin-1/2; IV, colocalization of Homer-1 and Synapsin-I/II without Pcdh19. (**f**) Localization of exogenously expressed Pcdh19 and mCherry in hippocampal neurons at DIV21. Pcdh19-EGFP signals colocalized with homer-1. Scale bar, 10 μm in (**a**–**c**,**f**) 2 μm in (**d**).

We next examined cellular localization of Pcdh19 in cortical and hippocampal neurons by immunofluorescence staining. In monolayer cultures of these neurons at DIV7 to DIV21, Pcdh19 was detected as punctate immunofluorescence signals on their dendrites, most of which are in contact with axons (Figs 3a–c and S3a,b). To determine whether these Pcdh19 puncta associate with synapses, we used hippocampal cultures at DIV21. In neurons in these cultures, some of the Pcdh19 signals colocalized with the post-synaptic marker Homer-1 (Fig. 3d, arrow-I and **3e**, graph-I), or both Homer-1 and the pre-synaptic marker Synapsin-1/2 (Fig. 3d, arrow-II and **3e**, graph-II). However, other Pcdh19-positive puncta colocalized with neither of these synaptic proteins (Fig. 3d, arrow-III and 3**e**, graph-III). We also found Pcdh19-negative puncta with both pre- and post-synaptic markers (Fig. 3d, arrow-IV and **3e**, graph-IV). Quantification of these signals indicated that about 25% of Pcdh19-positive puncta colocalize with either Homer-1 (12%) or Synapsin-1/2 (13%), and 4.5% colocalize with both of the synaptic proteins (90 puncta from three neurons were examined). The rest of the Pcdh19-positive puncta (∼70%) did not colocalize with these synaptic proteins. We also examined localization of exogenously expressed Pcdh19 in hippocampal neurons and found that, unlike the endogenous protein, most of the exogenously expressed Pcdh19 colocalized with Homer-1 at spine-like processes (Fig. 3f, arrows), as reported previously^19^. These results suggest that, although Pcdh19 is capable of localizing to spines or synapses in certain physiological or artificial conditions such as overexpression, this protein is not a constitutive component of synapses.

Previous studies showed that δ2 non-clustered Pcdhs interact with the WAVE complex through their cytoplasmic domain^7, 20–22^, and Pcdh17 generates unique unstable contacts between growing axons via this interaction^7^. We therefore compared the localization of Pcdh19 with that of the WAVE-complex protein Abi-1 (Supplementary Fig. S3c,d) using hippocampal cultures, and found that 57% of Pcdh19-positive puncta colocalized with Abi-1 (90 puncta from three neurons were examined). These results suggest that Pcdh19 also regulates cell-cell contacts at least partly through its interaction with the WAVE complex in these neurons.

### Neurite and spine morphology of neurons

**Figure 4:**
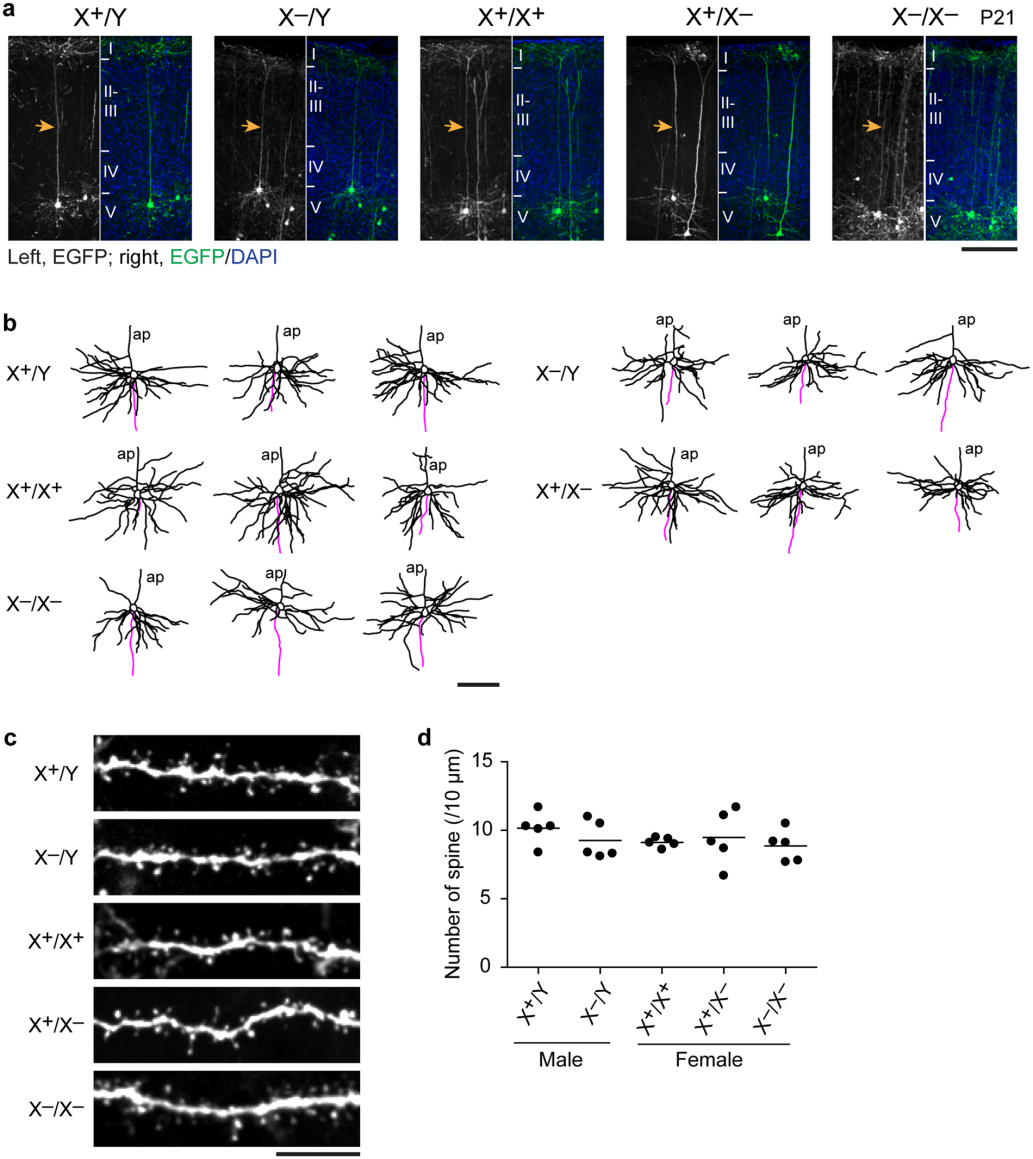
Formation of apical and basal dendrites and spines of layer-Va neurons in *Pcdh19* mutants. (**a**) Representative layer Va neurons labeled by *in utero* electroporation of GFP. Arrows indicate the apical dendrite of each neuron. Brains were fixed at P21. (**b**) Examples of dendritic branches of layer Va neurons. Tracings of dendrites and axons were shown in black and magenta, respectively. ap, apical dendrite. (**c**) Examples of spines along basal dendrites of layer Va neurons. (**d**) Number of spines per 10 μm along basal dendrites did not significantly differ among wild-type and *Pcdh19* mutant mice (*P* = 0.6184, n = 5 neurons). Scale bars, 100 μm in (**a**,**b**) 10 μm in (**c**). 100 μm-thick slices were used for analysis.

To test whether Pcdh19 plays a role in neurite organization, we examined neurite morphology of cortical layer Va neurons. To this end, we labeled neurons with GFP by *in utero* electroporation of this fluorescence probe. Each of the neurons labeled at layer Va extended a single axon, a single apical dendrite, and a number of basal dendrites whose branching pattern was highly variable among neurons (Fig. 4a). This characteristic neurite pattern was observed in all male and female genotypes and there were no detectable morphological differences among them (Fig. 4b). We also examined spines on basal dendrites, and found no obvious difference in their morphology (Fig. 4c) or numbers (Fig. 4d) between the genotypes. These results indicate that layer Va neurons were not affected in their gross anatomy by *Pcdh19* knockout. In addition, we found no defects in the cortico-cortical projection of axons through the corpus callosum (Supplementary Fig. S4), suggesting that *Pcdh19* mutants have normal axon projection at least in this pathway.

### Pcdh19 hemizygous male and heterozygous female mutants show normal anxiety-like behavior and social interaction

Next, we examined whether *Pcdh19* mutant mice show any behavioral defects, paying particular attention to sex-dependent differences. We performed comprehensive behavioral tests, using *Pcdh19* hemizygous male and heterozygous female mutants, in the sequence shown in Table 1. Since it is not possible to obtain all female genotypes (wild-type, heterozygous and homozygous) as littermates in our mating protocol (see Methods) and also human EFMR occurs exclusively in heterozygous mutants of the *PCDH19* gene, we compared only wild-type and heterozygous mutant mice in the female behavioral experiments.

**Table 1:** Phenotypes of *Pcdh19* hemizygous male (X^−^Y) and heterozygous female (X^+^X^−^) mutants compared to wild-type counterparts.

Test	Measure	Phenotype	
		Male	Female
1. General health/neurological screen (GHNS)	Whisker, coat, reflexes	n.s.	n.s.
	Body weight (g)	Decreased	n.s.
	Rectal temperature (°C)	n.s.	Decreased
	Grip strength (N)	Decreased	n.s.
	Wire hang (sec)	n.s.	Decreased
2. Light/Dark transition	Distance traveled in light (cm)	n.s.	Increased
	Distance traveled in dark (cm)	n.s.	Increased
	Stay time in light (sec)	n.s.	n.s.
	Stay time in dark (sec)	n.s.	n.s.
	Number of transitions	n.s	n.s.
	Latency to enter light (sec)	n.s	n.s.
3. Open field	Total distance (cm)	See Fig. S5	See Fig. S5
	Vertical activity	See Fig. S5	See Fig. S5
	Center time (sec)	See Fig. S5	See Fig. S5
	Stereotypic Counts	See Fig. S5	See Fig. S5
4. Elevated plus maze	Distance traveled (cm)	Decreased	Decreased
	Time on open arms (%)	n.s.	n.s.
	Number of entries	Decreased	n.s.
	Entries into open arms (%)	n.s.	n.s.
5. Hot Plate	Latency (sec)	Decreased	n.s
6. Social Interaction (Novel environment)	Total duration of contacts (sec)	n.s.	n.s.
	Mean duration per contacts (sec)	n.s.	n.s.
	Number of contacts	n.s.	n.s.
	Total duration of active contact (sec)	n.s.	n.s.
	Distance traveled (cm)	n.s.	n.s.
7. Rotarod	Latency to fall (sec)	n.s.	n.s.
8. Social interaction (Crawly’s version)	Total distance (cm)	n.s.	n.s.
	Average speed (cm/sec)	n.s.	n.s.
	Sociability	n.s.	n.s.
	Social preference	n.s.	n.s.
9. Prepulse inhibition	Acoustic startle response at 110 dB	n.s.	Increased
	Acoustic startle response at 120 dB	n.s.	n.s.
	Prepulse inhibition (%) at 110 dB	n.s.	n.s.
	Prepulse inhibition (%) at 120 dB	n.s.	n.s
10. Porsolt forced swim	Immobility (%)	See Fig. 5	See Fig. 5
	Distance traveled (cm)	Decreased	n.s.
11. T-maze spontaneous alternation	Correct responses (%), latency (sec) and distance traveled (cm) during training	See Fig. S6	See Fig. S6
	Correct responses in the delay session	See Fig. S6	See Fig. S6
12. Barnes maze	Distance (cm), latency (sec), search errors and omission errors	See Fig. S7	See Fig. S7
	Probe test 1 day after training	See Fig. S8	See Fig. S8
	Probe test 32 days after training	See Fig. S8	See Fig. S8
13. Tail suspension	Immobility (%)	See Fig. 5	See Fig. 5
	Distance traveled (cm)	Increased	Increased
14. Fear conditioning	Freezing (%) and distance traveled (cm) in	See Fig. 7	See Fig. 7
	2) context test 1 day and 28 days after conditioning.	See Fig. 7	See Fig. 7
	3) cued test with altered context 1 day and 28 days after conditioning.	See Fig. 7	See Fig. 7
15. Open field (2nd)	Total distance (cm)	See Fig. 6	See Fig. 6
	Vertical activity	See Fig. 6	See Fig. 6
	Center time (sec)	See Fig. 6	See Fig. 6
	Stereotypic Counts	See Fig. 6	See Fig. 6
16. Social interaction test in a home cage	Social interaction activity	n.s.	n.s.
	Locomotor activity	n.s.	n.s.

n.s., no statistical difference (*P* > 0.05); Increased, statistically increased (*<*0.05); Decreased, statistically decreased (<0.05).

In the light-dark transition, open field and elevated plus maze tests, which were used to assess anxiety-like behavior, there were no significant differences between wild-type and *Pcdh19* mutant animals except in some parameters measured (Table 1 and Supplementary Fig. S5), leading us to judge that anxiety-like behaviors essentially do not differ between the genotypes. We also found no significant differences in the social interaction between wild-type and mutants (Table 1).

### Pcdh19 hemizygous male and heterozygous female mutants have normal working and spatial memory

Since *Pcdh19* is expressed in the hippocampal CA region, we examined working and spatial memory of *Pcdh19* mutant mice by the T-maze and Barnes maze tests, respectively. Both *Pcdh19* male and female mutants showed no significant abnormalities in spontaneous and delayed T-maze tests (Fig. S6), suggesting their working memory is not affected. In the Barnes maze test, male and female mice received training over 18 days (Supplementary Fig. S7a–d) and 16 days (Supplementary Fig. S7e–h), respectively. Although there were significant differences in the number of search errors in males (Supplementary Fig. S7c) and the number of omission errors in females (Supplementary Fig. S7h) during the training, both genotypes in male and female mice finally acquired similar levels of spatial memory. In probe tests performed one day (Supplementary Fig. S8a–d) and 32 days (Supplementary Fig. S8e–h) after the last day of training, no significant differences were detected between *Pcdh19* hemizygous and wild-type male mice or between *Pcdh19* heterozygous and wild-type female mice. These results suggest that neither *Pcdh19* hemizygous male nor heterozygous female mutants have detectable abnormalities in the accuracy and retention of short- or long-term spatial memory.

### Pcdh19 hemizygous male and heterozygous female mutants show impaired activities under stress conditions

**Figure 5:**
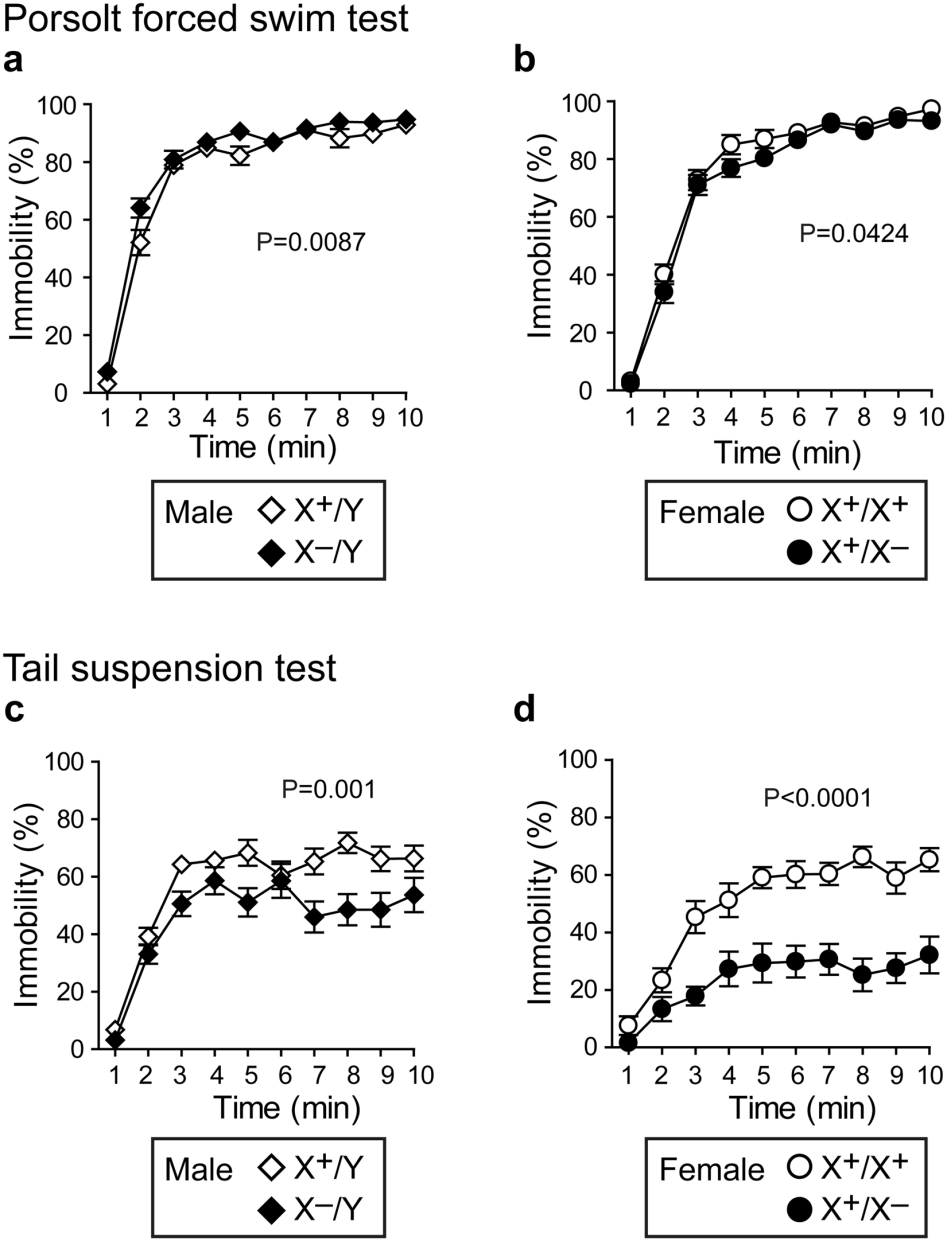
Altered behavior of *Pcdh19* mutants in the Porsolt forced swim and tail suspension tests. (**a**,**b**) Porsolt forced swim test with male (**a**) and female (**b**) mice. (**a**) *Pcdh19* hemizygous male mutants (X^−^Y) showed a significantly higher immobility rate (**a**) compared to wild-type male mice, whereas heterozygous female mutants (X^+^X^−^) showed a lower immobility rate than wild-type female mice (**b**). (**c**,**d**) Tail suspension test with male (**c**) and female (**d**) mice. Both *Pcdh1*9 hemizygous (X^−^Y) and heterozygous female (X^+^X^−^) mutants showed a significantly lower immobility rate compared to wild-type counterparts.

We examined depression-like behavior of mice by the Porsolt forced swim test. *Pcdh19* hemizygous males showed a significantly higher immobility rate (Fig. 5a) compared to wild-type males. By contrast, heterozygous females showed a lower immobility rate (Fig. 5b) than wild-type females. These results suggest that *Pcdh19* hemizygous males are slightly hypoactive, whereas heterozygous females are slightly hyperactive under stress conditions. We also examined their activity by the tail suspension test. Unlike in the Porsolt forced swim test, *Pcdh19* hemizygous males showed a lower immobility rate (Fig. 5c) than wild-type male mice. The heterozygous female mutants also showed a lower immobility rate (Fig. 5d) than wild-type female mice. Thus, although male mice showed opposite tendencies depending on the context of the stress, both *Pcdh19* hemizygous males and heterozygous females were abnormal in their mobility under stress conditions.

**Figure 6:**
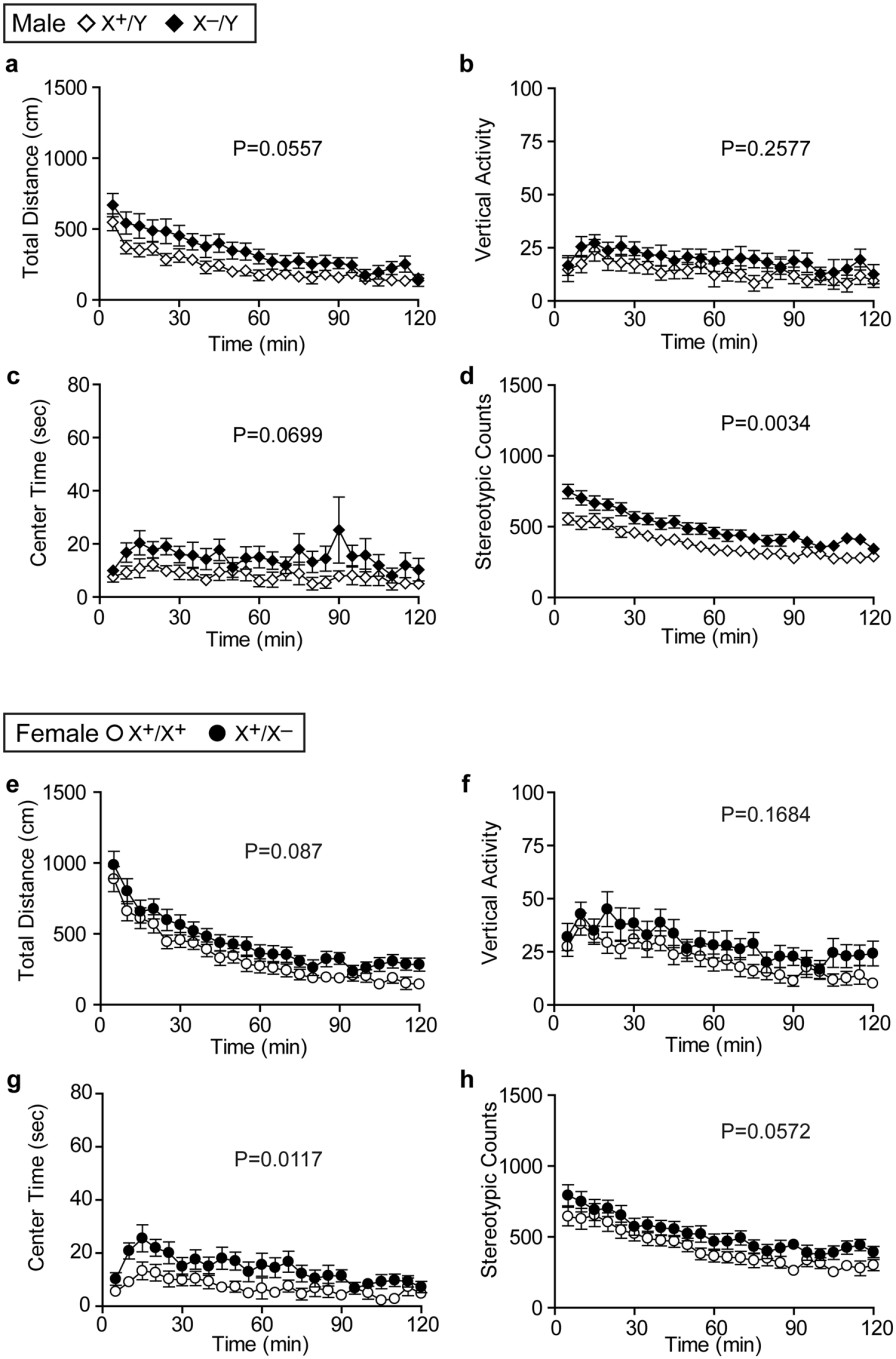
Increased activity of *Pcdh19* mutant mice in the second open field test. (**a**–**d**) Test with male mice. (**e**–**h**) Test with female mice. Total distance (**a**,**e**), vertical activity (**b**,**f**), time spent in the center of box (**c**,**g**), and count of stereotypic activity (**d**,**h**) were measured. *Pcdh19* hemizygous males showed significantly increased stereotypic counts, whereas *Pcdh19* heterozygous females showed significantly increased time spent in the center of the box compared to wild-type counterparts.

We thought a possibility that the behavior of mutant mice might change through aging. To test this, we performed the second open field test in the latter part of the behavioral battery at the age of 34 weeks (Table 1). In this test, we found that *Pcdh19* mutant mice tended to show higher values than wild-type counterparts in every parameter (Fig. 6), whereas such tendencies were not observed in the first round of the open field test performed at the age of 11–12 weeks (Supplementary Fig. S5). *Pcdh19* hemizygous males and heterozygous females also tended to show hyperactivity in the social interaction test in a home cage, although they were not statistically significant (Table 1 Locomotor activity: male, p = 0.0693; female, p = 0.0782). These results are consistent with the idea that both *Pcdh19* hemizygous male and heterozygous female mutants become more hyperactive than wild-type counterparts by aging.

### Pcdh19 heterozygous female mice showed decreased fear responses

**Figure 7:**
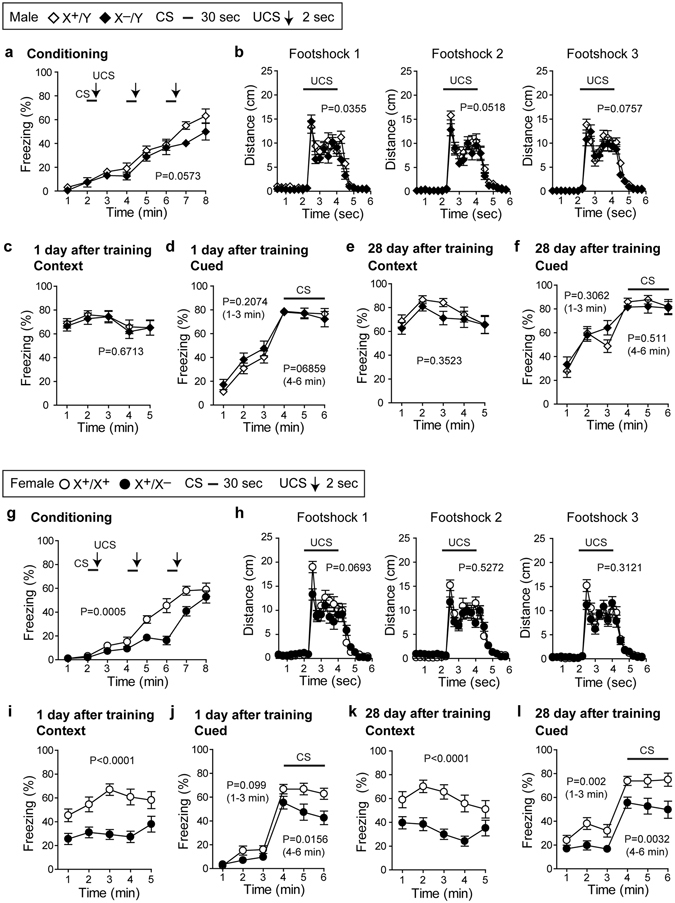
*Pcdh19* hemizygous male and heterozygous female mutants show differential fear responses. Fear conditioning tests with male (**a**–**f**) and female (**g**–**l**) mice. For fear conditioning, a cue signal (CS) of 30 sec followed by 2-sec unconditioned stimulus (UCS, foot shock) was applied at 2, 4 and 6 mins after starting the test. (**a**,**b**,**g**,**h**) In both wild-type and *Pcdh19* mutant males, the extent of freezing (**a**) and travel distance (**b**) induced by unconditioned stimuli (three footshocks) was increased. (**c**,**d**) Context-dependent (**c**) and cued (**d**) fear responses did not differ between wild-type and *Pcdh19* hemizygous mutant males. (**e**,**f**) Retention test held 28 days after the training for contextual (**e**) and cued (**f**) fear responses in male mice. There were no significant differences between wild-type and *Pcdh19* hemizygous mutant males. (**g**,**h**) In both wild-type and *Pcdh19* heterozygous mutant females, the extent of freezing (**g**) and travel distance (**h**) induced by unconditioned stimuli (three footshocks) was increased, but with a significantly reduced freezing response in the mutants. (**i**,**j**) Contextual (**i**) and cued (**j**) fear responses significantly decreased in *Pcdh19* heterozygous mutant females. (**k**,**l**) Retention test held 28 days after the training for contextual (**k**) and cued (**l**) fear responses. There was a significant decrease in both responses in *Pcdh19* heterozygous mutant females compared to wild-type mice. In the experiments in (**c** and **i**), mice were put in the same box as used for the conditioning, whereas in (**d** and **j**), mice were put in a box different from the one used for the conditioning. The cue signal was applied at 4 mins after starting the test and lasted for 2 mins.

We also performed the fear-conditioning test to examine associative fear learning and memory in *Pcdh19* mutants. After conditioning by contextual and un-contextual cues (male in Fig. 7a,b and female in Fig. 7g,h), fear responses were tested 1 day (male in Fig. 7c,d and female in Fig. 7i,j) and 28 days (male in Fig. 7e,f and female in Fig. 7k,l) after the conditioning. In the test held one day after the conditioning, *Pcdh19* hemizygous male mutants showed no significant difference in contextual (Fig. 7c) or cued (Fig. 7d) fear responses from wild-type males. There was also no significant difference between these mice in the test performed 28 days after the last day of conditioning (Fig. 7e,f). By contrast, *Pcdh19* heterozygous female mutants showed a lower fear response in the conditioning process (Fig. 7g) and also showed lower contextual (Fig. 7i) and cued (Fig. 7j) fear responses in the test held one day after conditioning compared to wild-type female mice. These decreased contextual and cued fear responses were also observed at 28 days after conditioning (Fig. 7k,l). It is of note that the contextual fear response was particularly low in these female mutants (Fig. 7i,k). Thus, lack of *Pcdh19* affected fear conditioning only in heterozygous female mutants.

## Discussion

Our histological observations of *Pcdh19* mutant mice did not detect any gross changes in mutant brains, consistent with a recent observation^19^. We also failed to observe any cytological defects in mutant neurons, that is, dendrite patterning and spine morphology looked unchanged in layer Va neurons of *Pcdh19* mutants, despite the localization of Pcdh19 proteins to these structures. More detailed comparisons between Pcdh19–positive and –negative neurons, however, would be necessary to confirm these observations by future studies. On the other hand, comprehensive behavioral tests revealed that the mutant mice have behavioral abnormalities. Both *Pcdh19* heterozygous female and hemizygous male mutants were abnormal in several tests, suggesting that Pcdh19 is important in regulation of mouse behavior, regardless of its sex, although anatomical basis of such behavioral defects remains to be further studied. A recent study reported that *Pcdh19* is involved in columnar distribution of neurons in the zebrafish tectum^6^, supporting the idea of its participation in neuronal functions. Importantly, we identified female-specific behavioral defects in *Pcdh19* mutant mice, that is, only heterozygous females displayed decreased fear responses. Since we did not examine the behavioral phenotypes of homozygous female mice, it remains to be determined whether the phenotypes found in the females were specific to the heterozygous female mutants. Even so, our results provide evidence that certain phenotypes are more severe in the female than the male mutants, suggesting that female-specific abnormalities observed in human EFMR were at least in part recapitulated in our mouse model.

Previous studies proposed a ‘cell interference’ hypothesis to explain why only heterozygous females are affected by *Pcdh19* mutations in humans^10, 14^. According to the hypothesis, the co-existence of Pcdh19–positive and –negative cells may affect neuronal interactions. Our observations confirmed that the heterozygous female cortex is indeed a mosaic of Pcdh19–positive and –negative cells at layer Va. Additionally, we showed that overexpression of Pcdh19 induced ectopic clustering of neurons, which is a typical act of homophilic adhesion molecules including protocadherins^3–5^. We suspect that such actions of Pcdh19 might be implicated in the phenotypes of heterozygous females. In wild-type cortices, Pcdh19 molecules were evenly expressed across the layer Va region. In cortices with mosaic Pcdh19 expression, however, Pcdh19-positive neurons locally remained. Such neurons might overly aggregate via Pcdh19s, as they lost neighbors that could counteract their excessive aggregation via mutual adhesive interactions. These putative processes might interfere with the normal network formation by these neurons, as proposed by the cell interference model.

This interference model is based on the assumption that Pcdh19 is not essential for brain functions in humans, as male mutants are not affected by *PCDH19* mutations unless mosaic mutations occur^12–14^. In the case of mice, however, Pcdh19 seems to have sex-independent functions. This implies that the phenotypes observed in heterozygous female mice might have been a mix of phenotypes brought about by cell interference and loss of function.

Epilepsy caused by *PCDH19* gene mutation in humans has an infantile onset, suggesting the possibility that *PCDH19* might be involved in brain development. Our results using mice showed that *Pcdh19* is expressed in developing as well as adult brains. It is, therefore, not clear whether the behavioral abnormalities in *Pcdh19* mutant mice were derived from developmental or physiological defects. Our behavioral analysis showed that *Pcdh19* mutants became more hyperactive in the second round of the open field test than in the first test, in which the second test was performed 22–23 weeks after the first test. This suggests that lack of *Pcdh19* may cause progressive abnormalities in aged brains. However, since the second open field test and the following social interaction test were performed after other behavioral tests, we cannot exclude the possibility that prior test experiences for mice could also have influenced on their behavior, making the mutant mice hyperactive in these tests. In addition, the hyperactivity of *Pcdh19* mutants became prominent in stress conditions. This stress-induced hyperactivity, rather than defects in memory, might have caused lower fear responses of the heterozygous mutants in the fear-conditioning test. Even so, it is important that this reduced fear response was only observed in heterozygous mutants. To elucidate how the mouse phenotypes observed in the present study are related to human EFMR is an important subject for future study.

## Methods

### Mice

The experiments using mice were performed in accordance with the protocol(s) approved by the Institutional Animal Care and Use Committee of RIKEN Center for Developmental Biology (RIKEN Kobe Branch). Behavioral experiments were approved by the Institutional Animal Care and Use Committee of Fujita Health University. The day of detection of the vaginal plug was defined as E0.5. To produce *Pcdh19* knockout mice (Accession No. CDB0839K: http://www2.clst.riken.jp/arg/mutant%20mice%20list.html), we used a target vector in which the whole first exon after the start codon of the *Pcdh19* gene was replaced by a *LacZ-neo* selection cassette (Supplementary Fig. S2a). The selection cassette was then removed using a *Sox-2*::*Cre* transgenic mouse (Jackson Laboratory) to produce a *Pcdh19* null mouse for phenotypic analysis except for X-gal staining. Mice were backcrossed with the C57BL/6N line in F8 generations. Genotyping of *Pcdh19* mutant mice was performed by PCR with the following primers (Supplementary Fig. S2b): P1, 5′-TGACCACCTGTTTGAGATCGATCCGTCCAG-3′; P2, 5′-GAGTTAACTATTGCCTACCCCACACACATG-3′; P3, 5′-AAGGGTTCCGGATCCCCATCAAGCTTATCG-3′. The wild-type allele (712 bp) was detected using P1 and P2 primers, and the *Pcdh19* mutant allele (450 bp) was detected using P2 and P3 primers. Sex of embryos and neonatal mice was confirmed by PCR using primers 5′-TCTAGAGAGCATGGAGGGCCATGT-3′ 5′-ATGCCACTCCTCTGTGACACTTTA-3′ for the male-specific marker *Sry* on the Y chromosome. For behavioral experiments of *Pcdh19* mutant mice, wild-type male (X^+^/Y) and *Pcdh19* heterozygous female (X^+^/X^−^) mice were crossed to obtain wild-type male (X^+^/Y) and female (X^+^/X^+^), hemizygous male (X^−^/Y) and heterozygous female (X^+^/X^−^) mice. For histological analysis, mice obtained by the above mating protocol were also compared with *Pcdh19* homozygous female mutants (X^−^/X^−^) at the same age, which were obtained from another pair of *Pcdh19* hemizygous male (X^−^/Y) and heterozygous female (X^+^/X^−^) mice, whose possible offspring are X^+^/Y, X^−^/Y, X^+^/X^−^ or X^−^/X^−^.

### Immunohistochemistry

Mice were deeply anaesthetized with Pentobarbital and the brain was fixed by cardiac perfusion of 4% paraformaldehyde (PFA) in PBS. Then, the brain was dissected out and incubated in 4% PFA in PBS for 10 hrs at 4 °C. The procedure for immunohistochemistry of cryo-sections was as previously described^7^. Briefly, fixed brains were immersed in 30% sucrose and frozen in OCT compound (Sakura Finetek). Sections were cut at 10 μm thickness with a Microm HM-500 M Cryostat (GMI). For Pcdh19 immunostaining, sections were incubated in HistoVT One (Nacalai) at 90 °C for 20 min to retrieve antigens. Sections were permeabilized with 0.1% Triton X-100 in PBS (PBST) and then incubated in 3% BSA in PBST for blocking. The sections were incubated in the blocking solution containing primary antibody for 2 hrs at room temperature (RT) or overnight at 4 °C. Then the sections were incubated in the blocking solution contacting fluorochrome-conjugated secondary antibody for 1 hr at RT. The fluorochromes used were Alexa Fluor 488, 568, 647 (Invitrogen), cy3 or cy5 (Jackson ImmunoResearch). The sections were mounted on MAS-coated glass slides (Matsunami) using FluorSave reagent (Calbiochem). For analysis of GFP-labeled layer Va neurons in the somatosensory cortex, fixed brains were sliced 100 μm thick with a tissue slicer (Dosaka). The slices were incubated with primary antibody followed by secondary antibody in PBS containing 0.1% Tween overnight at 4 °C. Images were acquired using a laser confocal microscope (IX71, Olympus) equipped with a spinning-disc unit CSU-X1 (Yokogawa), through the following objective lenses: MPlanFL N 2.5×/0.08 NA (callosal projection), UPlanSApo N 10×/0.40 NA (overall morphology of layer Va neurons), UPlanSApo 30 × S/1.05 NA (morphology of basal dendrites) and UPlanSApo 60 × S2/1.30 NA (spine morphology) objective lenses (Olympus). A scientific Neo CMOS camera (Andor) was used to acquire images, and these were processed using MetaMorph software (Molecular Devices) and then analyzed with ImageJ software (http://imagej.nih.gov/ij/). To analyze the morphology of neurites and spines of EGFP-labeled neurons, stack images were processed using maximum intensity projection. To compare the morphology of layer-Va neurons, dendrites and axons of labeled neurons were manually traced with drawing tools. To measure the number of spines in layer-Va neurons in a projected image, a region along their basal dendrites, at a distance of about 30–80 μm from the somata, was randomly selected, and the number of dendrites in the region was counted. Data were analyzed by one-way ANOVA using GraphPad Prism 4 software.

### Antibodies

Pcdh19 antibody was raised by immunizing a rabbit with the GST-fused cytoplasmic region of Pcdh19 (Val1041-Leu1140 a.a.) and subsequently affinity-purified with the antigen. A GST-reactive population was also removed. The following antibodies were purchased: rat anti-Ctip2, chicken anti-LacZ and chicken anti-GFP (Abcam); mouse anti-Homer1 and guinea pig anti-Synapsin1/2 (Synaptic systems); rabbit anti-MAP2, mouse anti-Tau1 and guinea pig anti-Vglut2 (Millipore); rabbit anti-FLAG and mouse anti-MAP2 (Sigma-Aldrich); and mouse anti-Abi-1 and rabbit anti-RFP (MBL).

### *In situ* hybridization

*Pcdh19* mRNA was detected by *in situ* hybridization as described previously^23^. Mouse *Pcdh19* cDNA was cloned from the Mouse Brain Marathon-Ready cDNA library (Takara). The template for probe synthesis (positions 3692–4678 of the *Pcdh19* gene sequence GenBank Acc.No. NM_001105245) was amplified by PCR using the primers 5′-TCTGCCCTTGTCCTAATATACCTGTCCCCA-3′ and 5′-TGACTTGGAGCGGGTGGGCATGGGATTCCG-3′. The DIG-labeled antisense and sense RNA probes were synthesized *in vitro* using a DIG RNA labeling kit (Roche). E18.5 and P7 brains were cryo-sectioned at 14 μm thickness.

### In utero electroporation

*In utero* DNA transfer to the cerebral cortex by electroporation was performed as previously described^24^. Briefly, for overexpression of Pcdh19, 1–2 µl of PBS containing 0.5 mg/ml of pNeuroD-Cre (a kind gift from Y. Tanabe), 0.5 mg/ml of pCAG-loxP-neo-pA-loxP-GFP (pCALNL5-GFP) and 0.5 mg/ml control vector (pCALNL5) or pCALNL5-Pcdh19-FLAG were introduced into the ventricle of the brain at E12.5. The original pCALNL5 vector was a kind gift from I. Saito. Five 50-msec pulses of 35 volts were delivered to the embryonic heads at 950-msec intervals using electrodes (LF650P3, BEX) connected to an electroporator (BEX CUY21). The brains were fixed at E18.5. In the experiment for labeling layer Va neurons, 0.2 mg/ml of pCAG-ERT2creERT2 (Addgene 13777) and 1 mg/ml of pCALNL-GFP were introduced into the ventricle of the brain at E13.5. Newborn mice were transferred to host ICR female mice and tamoxifen (Sigma-Aldrich) dissolved in corn oil (Sigma-Aldrich) was administrated at P10–12 by oral gavage to the host mice (5 mg per animal). The electroporated mice were sacrificed at P21 to fix their brains.

### X-gal staining

Brains were fixed in PBS solution containing 4% PFA and 0.2% glutaraldehyde for ∼10 hrs at 4 °C and treated with X-gal staining buffer containing 2 mM MgCl_2_, 0.01% sodium deoxycholate, 0.02% Nonidet P-40, 5 mM potassium ferricyanide, 5 mM potassium ferrocyanide and 1 mg/ml X-gal (Nacalai tesque) in PBS for ∼8 hours at RT in the dark.

### Hippocampal and cortical neuronal culture

Cultures of dissociated hippocampal and cortical neurons were performed as previously described^7^. Briefly, hippocampal and cerebral cortical regions were dissected from E17.5 embryos and incubated with a solution containing 10 U/ml Papain (Nacalai tesque) for 20 min at 37 °C. After cells were dissociated and gently centrifuged, neurons were re-suspended in NeuroBasal medium (Thermo fisher) with B27 supplements (Thermo fisher), and plated on 12 mm-coverslips, which are coated with 1 mg/ml poly-D-lysine followed by 1 μg/ml laminin, at a cell density of ∼7,500/cm^2^. The cultured neurons were fixed with 2% PFA in Hanks’ balanced salt solution containing 1 mM Ca^2+^, Mg^2+^ (HBSS) and 4% Sucrose for 20 mins at RT, and immunostained with primary and secondary antibodies described above. Images were acquired with an epi-fluorescence microscope (Axioplan2, Carl Zeiss, Inc.) equipped with a cooled CCD camera (ORCA-R2; HAMAMATSU) and Plan Apochromat 63x/1.40 NA objective lens. To determine the localization of Pcdh19 and Abi-1 along dendrites, ImageJ software was used as follows: a region containing clearly distinguishable fluorescent clusters in each image was selected and a threshold of fluorescent intensity was manually set so that the threshold matched the margin of these clusters. Then, we applied the ‘Particle analysis’ Plugin to the whole image to detect all clusters whose fluorescent intensity was above the threshold.

### Preparation of brain samples for Western blotting

Whole brain and cortex, including hippocampus, were collected from male E10.5 and E14.5 embryos, respectively. Cortex and hippocampus were separately collected from male E17.5, P2, P7, P14, P21, P28 and adult (2-month-old) and lysed in PBS containing 0.1% Nonidet P-40 and a protease inhibitor cocktail (Roche). The lysates were then mixed with a sample buffer and subjected to SDS-PAGE. Proteins were transferred to PVDF membranes (Millipore) using a wet-tank system (BioRad). The primary antibodies were rabbit anti-Pcdh19 and mouse anti-α-tubulin (DM1A, Sigma-Aldrich), and the secondary antibodies were donkey anti-rabbit IgG and sheep anti-mouse IgG (GE healthcare). Detection was performed using Western Lightning ECL Pro (PerkinElmer) and ImageQuant LAS 4000 (GE Healthcare).

### Behavior tests

Comprehensive behavioral tests were performed as previously described^25^. Briefly, the tests were carried out with a male pair of wild-type and *Pcdh19* hemizygous mutant and a female pair of wild-type and *Pcdh19* heterozygous mutant mice. Male and female mice were separately housed, and each cage contained two wild-type and two mutant mice. n = 17–20 mice per genotype were used in each test. Experiments were performed in the sequence as shown in Table 1. The first test started at 10 weeks for each sex. Behavioral testing was performed between 9:00 a.m. and 6:00 p.m. For automatic acquisition of behavioral data, the applications based on the public domain Image J programs, which was modified by Tsuyoshi Miyakawa for each test, was used (available through Ohara & Co.). Data are presented as mean ± S.E.M. Statistical analysis was performed using StatView software (SAS Institute, Cary, NC). Data were analyzed by two-tailed *t*-test, paired t-test, one-way ANOVA, two-way ANOVA or two-way repeated measures ANOVA. P-values below 0.05 were considered as statistically significant.

## Electronic supplementary material

Supplementary Information
