# Supplementary material for: Loss of X-linked Protocadherin-19 differentially affects the behavior of heterozygous female and hemizygous male mice
Source: Sci Rep. 2017 Jul 19;7:5801. doi: 10.1038/s41598-017-06374-x (PMC5517645; doi:10.1038/s41598-017-06374-x)
Supplement: Supplementary file 1 — Supplementary Information [file 41598_2017_6374_MOESM1_ESM.pdf]

Supplementary information

Loss of X-linked *Protocadherin-19* differentially affects the behavior of heterozygous female and hemizygous male mice

Shuichi Hayashi, Yoko Inoue, Satoko Hattori, Mari Kaneko, Go Shioi, Tsuyoshi Miyakawa, and Masatoshi Takeichi

Figure S1

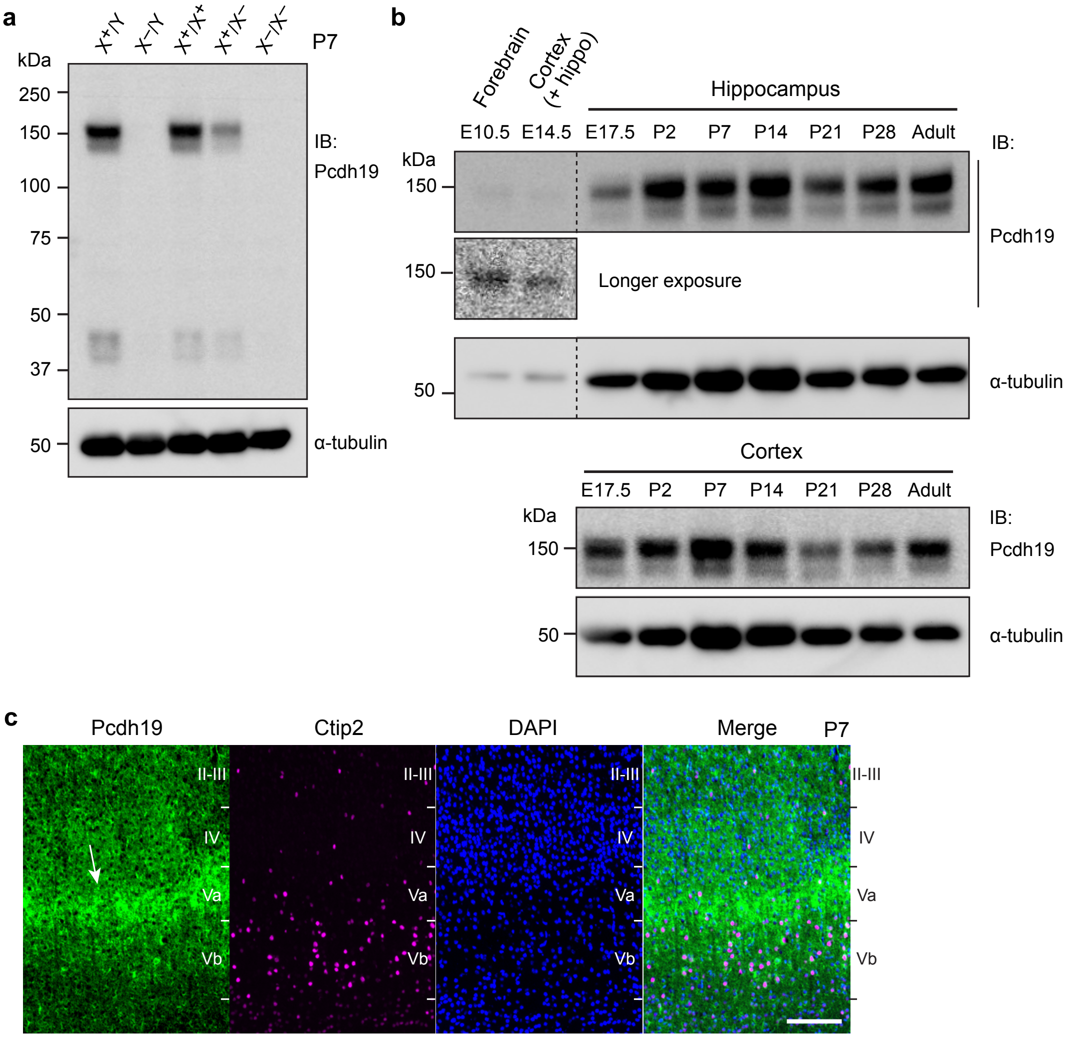

Supplementary Fig. S1. Developmental expression of *Pcdh19* protein in the cerebral cortex and hippocampus.

(**a**) Western blots to detect Pcdh19 proteins in the lysates of cerebral cortex from different genotypes of *Pcdh19* at P7. The antibody recognizes a ~150kDa protein in wild-type (X^+Y, X^+X^+) and heterozygous mutant (X^+X^-), which disappears in *Pcdh19* null mutants (X^-Y, X^-X^-). (**b**) Expression of Pcdh19 in the forebrain at E10.5, cortex including hippocampal formation at E14.5 and the hippocampus from E17.5 to adult (upper panels), and in the cerebral cortex from E17.5 to adult (lower panels). α-tubulin was used for a loading control. (**c**) Distribution of Pcdh19 protein in the somatosensory cortex at P7. It mainly localized in layer Va. Anti-Ctip2 antibody was used as a marker for layer Vb neurons. Scale bars, 100 μm. 10 μm-thick sections were used.

Figure S2

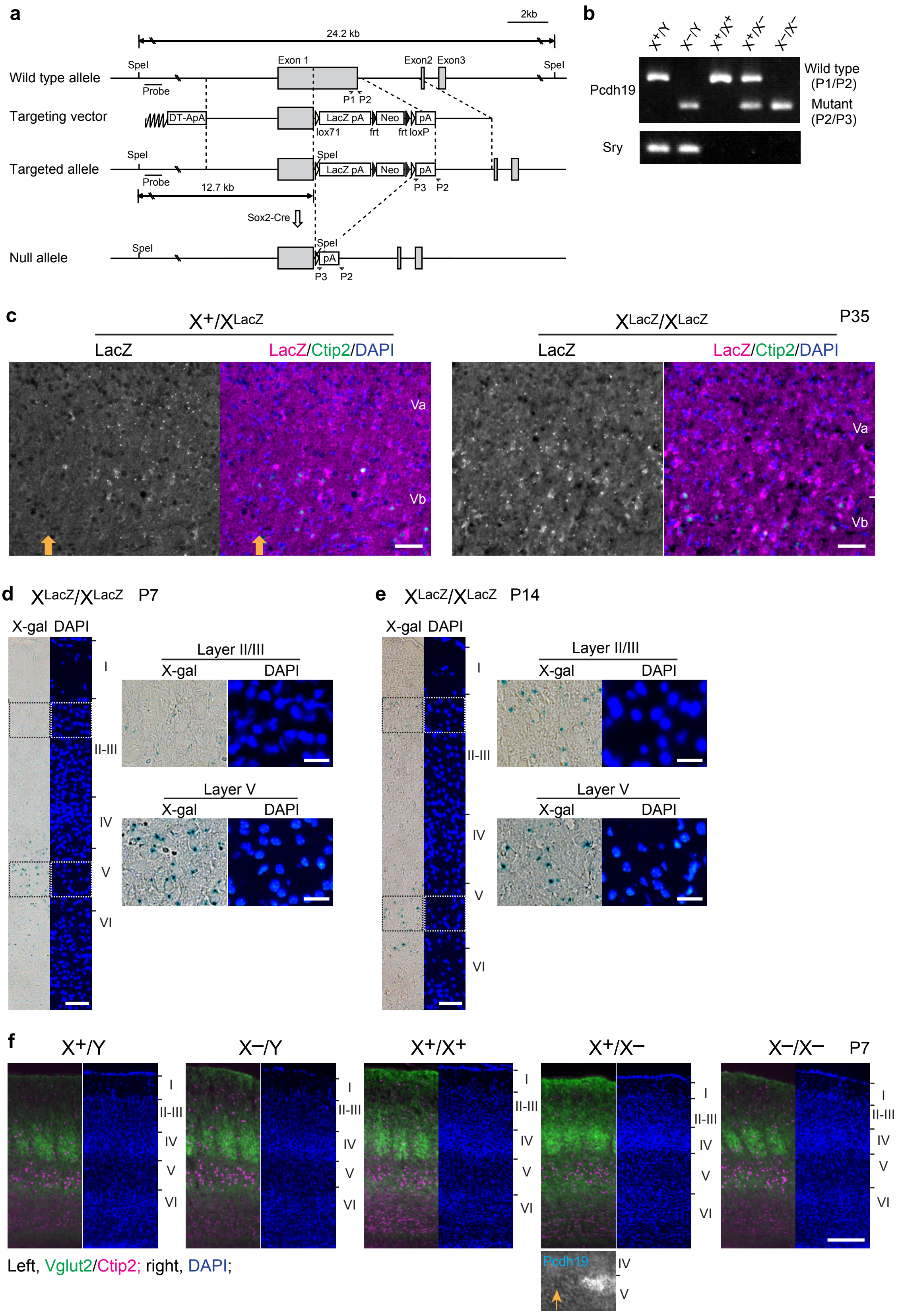

Left, Vglut2/Ctip2; right, DAPI;

Supplementary Fig. S2. **Generation of *Pcdh19* knockout mouse.**

(**a**) Strategy of homologous recombination for targeting the *Pcdh19* gene. The exons are depicted by grey boxes. The whole first exon after the start codon was replaced with a *LacZ-neo* selection cassette. The *Pcdh19* knockout/*LacZ*-knockin mouse was then crossed with the mouse carrying *Sox-Cre* to remove the *LacZ*-cassette to generate a *Pcdh19* null mutant mouse line. (**b**) Genotyping of mice by PCR with specific primers for the *Pcdh19* gene and the *loxP*-pA region of the *LacZ-neo* cassette. Primers for *Sry* on the Y-chromosome were used to distinguish male from female. (**c**) *LacZ* staining of the somatosensory cortex in *Pcdh19* heterozygous (left) and homozygous (right) mutants at P35. The left panel shows a typical example of mosaic expression of LacZ in layer V of the heterozygous mutant cortex, in which arrows indicate a region with fewer LacZ-positive cells. In the homozygous cortex at the right, LacZ signals are evenly distributed in layer V. (**d, e**) X-gal staining of the somatosensory cortex in *Pcdh19* homozygous mutants at P7 (**d**) and P14 (**e**). In addition to layer V, X-gal signals are weakly detected in layers II/III at both P7 and P14. (**f**) Somatosensory cortex of wild-type and *Pcdh19* mutants was stained for Vglut2, Ctip2 and DNA to visualize the barrel structure in layer IV, layer Vb and whole layer structures, respectively. There was no detectable difference in layer formation between wild-type and *Pcdh19* mutant mice. Mosaic Pcdh19 expression in the heterozygous female cortex was also shown by immunostaining for Pcdh19 proteins. Arrow points to a Pcdh19-negative zone. Images of female wild-type and heterozygous mice were taken from the same sections as those in Fig. 1i. 10 μm sections were used. Scale bars, 50 μm in c and left panels of d and e; 20 μm in right panels of d and e; 100 μm in f.

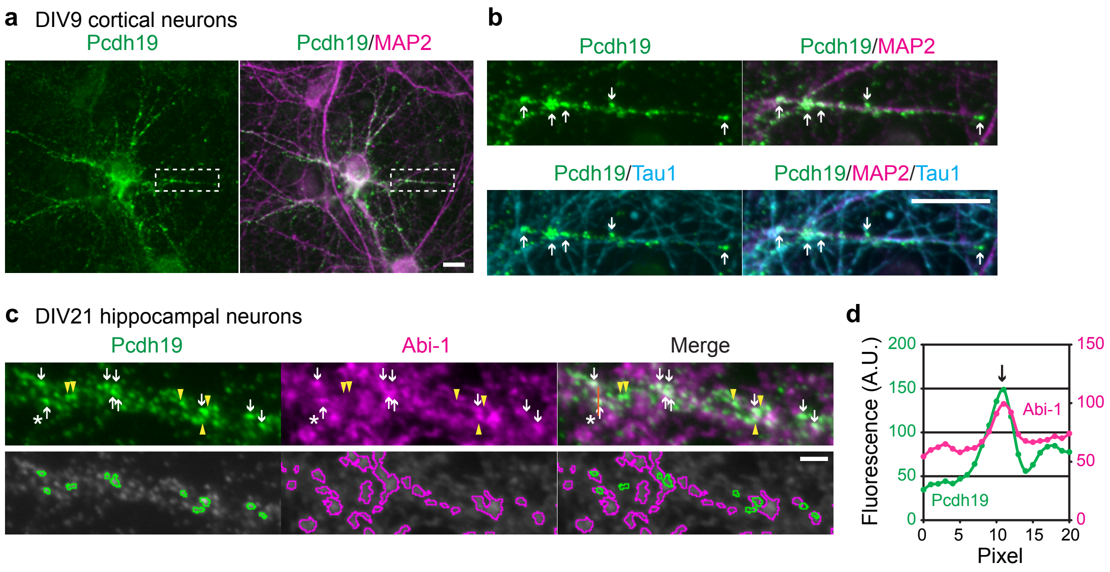

Supplementary Fig. S3 Localization of Pcdh19 and Abi-1 on dendrites of cortical and hippocampal neurons in cultures.

(**a**, **b**) Pcdh19 localization on cortical neurons at DIV9. The signals are detected along dendrites, which overlap with axons. The boxed region in **a** is enlarged in **b**. Arrows indicate representative Pcdh19-positive puncta. (**c, d**) Localization of Pcdh19 and Abi-1 on dendrites of hippocampal neurons at DIV21. White arrows and yellow arrowheads indicate colocalization and non-colocalization of Pcdh19 with Abi-1, respectively. Upper and lower panels show color and grey images with outlines of the puncta analyzed, respectively. (**c**) Fluorescence signals of Pcdh19 (green) and Abi-1 (magenta) at the points indicated by asterisks. Fluorescence intensities along the orange line in the upper merged image was measured. Scale bars, 10 μm in **a**, **b**; 2 μm in **c.**

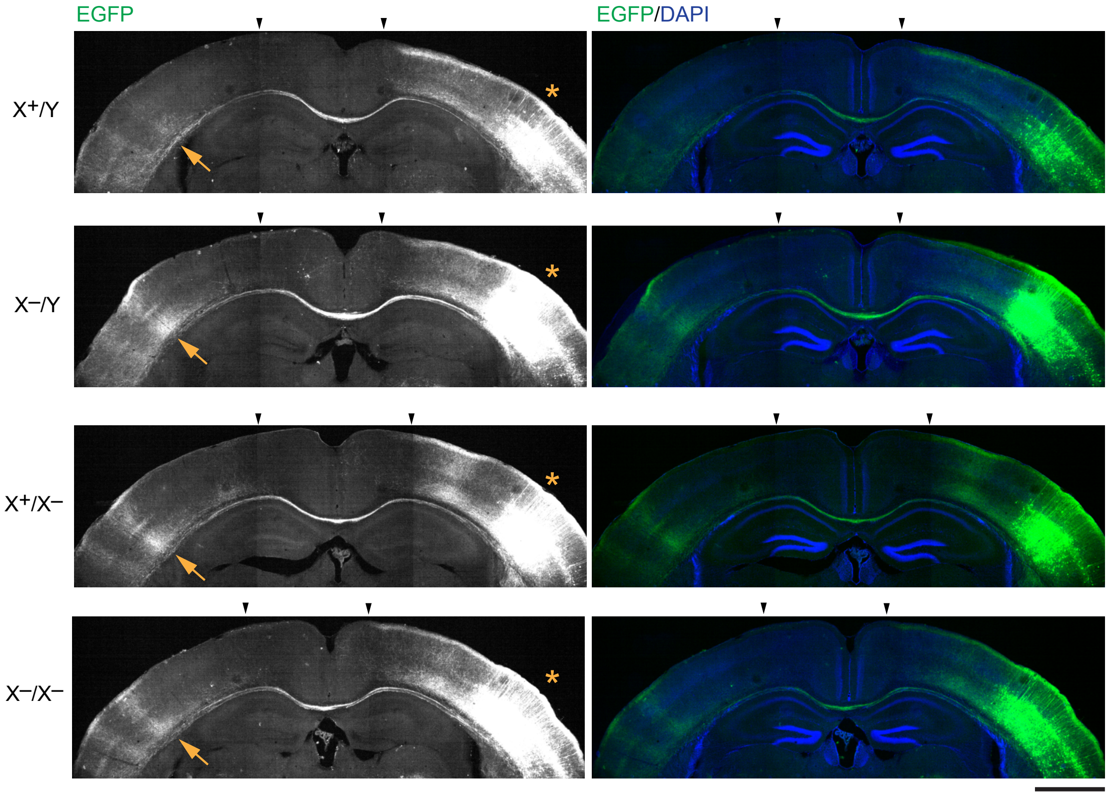

Supplementary Fig. S4 Normal callosal projection in *Pcdh19* mutants.

GFP was electroporated in the somatosensory cortex and callosal projection was observed at P21. Arrows indicate axon innervation into the contra-lateral side of the cerebral cortex. Callosal axons normally project into the opposite side of the somatosensory cortex in *Pcdh19* mutants. Three images were combined at the point indicated by arrowheads to obtain a single image. Scale bar, 1 mm.

Figure S5

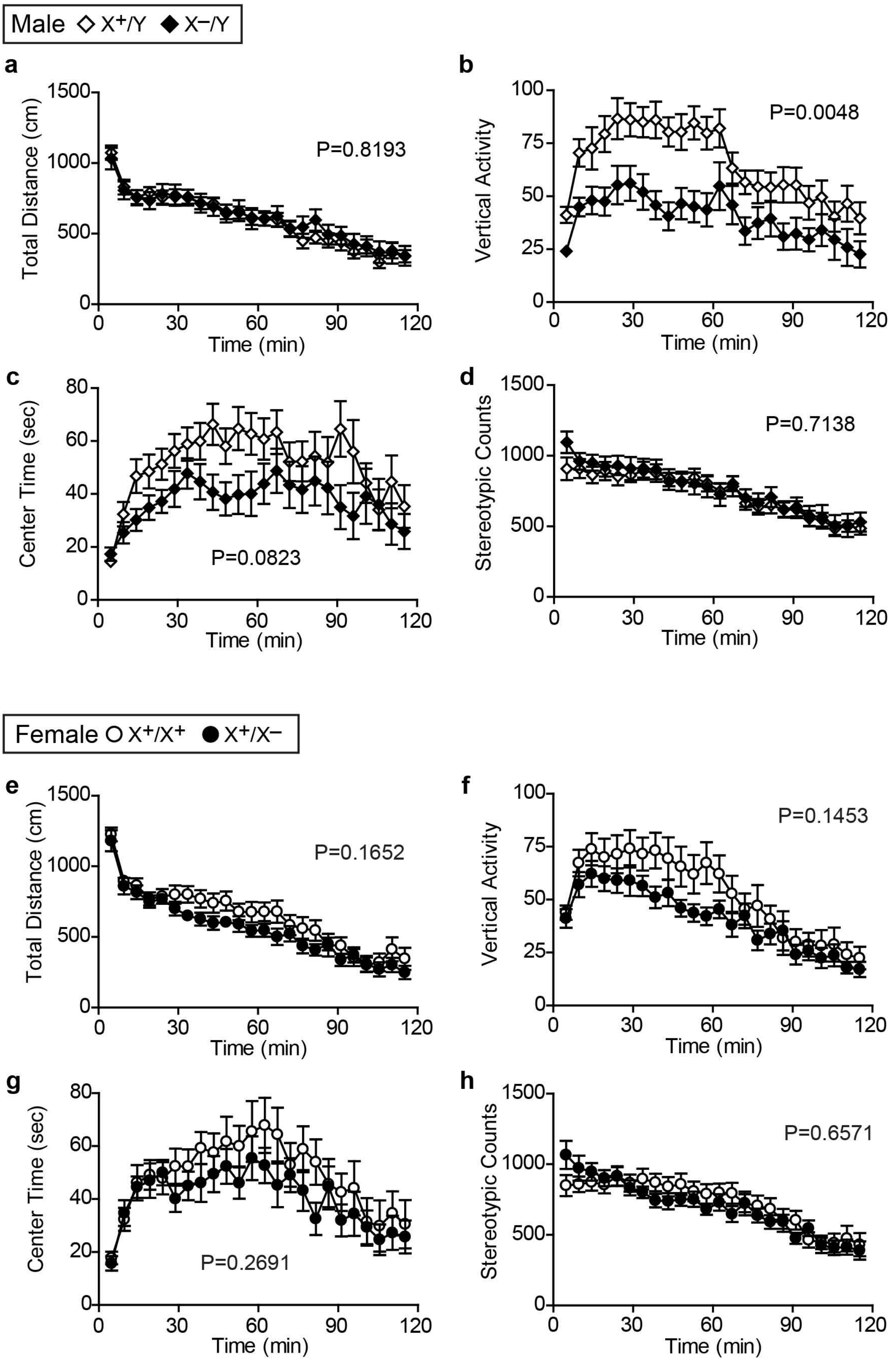
Male ◇ X+/Y ◆ X−/Y
a
Total Distance (cm)
Time (min)
P=0.8193
b
Vertical Activity
Time (min)
P=0.0048
c
Center Time (sec)
Time (min)
P=0.0823
d
Stereotypic Counts
Time (min)
P=0.7138
Female ○ X+/X+ ● X+/X−
e
Total Distance (cm)
Time (min)
P=0.1652
f
Vertical Activity
Time (min)
P=0.1453
g
Center Time (sec)
Time (min)
P=0.2691
h
Stereotypic Counts
Time (min)
P=0.6571

Supplementary Fig. S5 First open field test.

(**a-d**) Test with male mice. (**e-h**) Test with female mice. Total distance (**a, e**), vertical activity (**b**, **f**), time spent in center of box (**c, g**) and count of stereotypic activity (**d, h**) was measured. There were no significant differences between wild-type and *Pcdh19* mutants in males or females except for the lower vertical activity in mutant males.

Figure S6

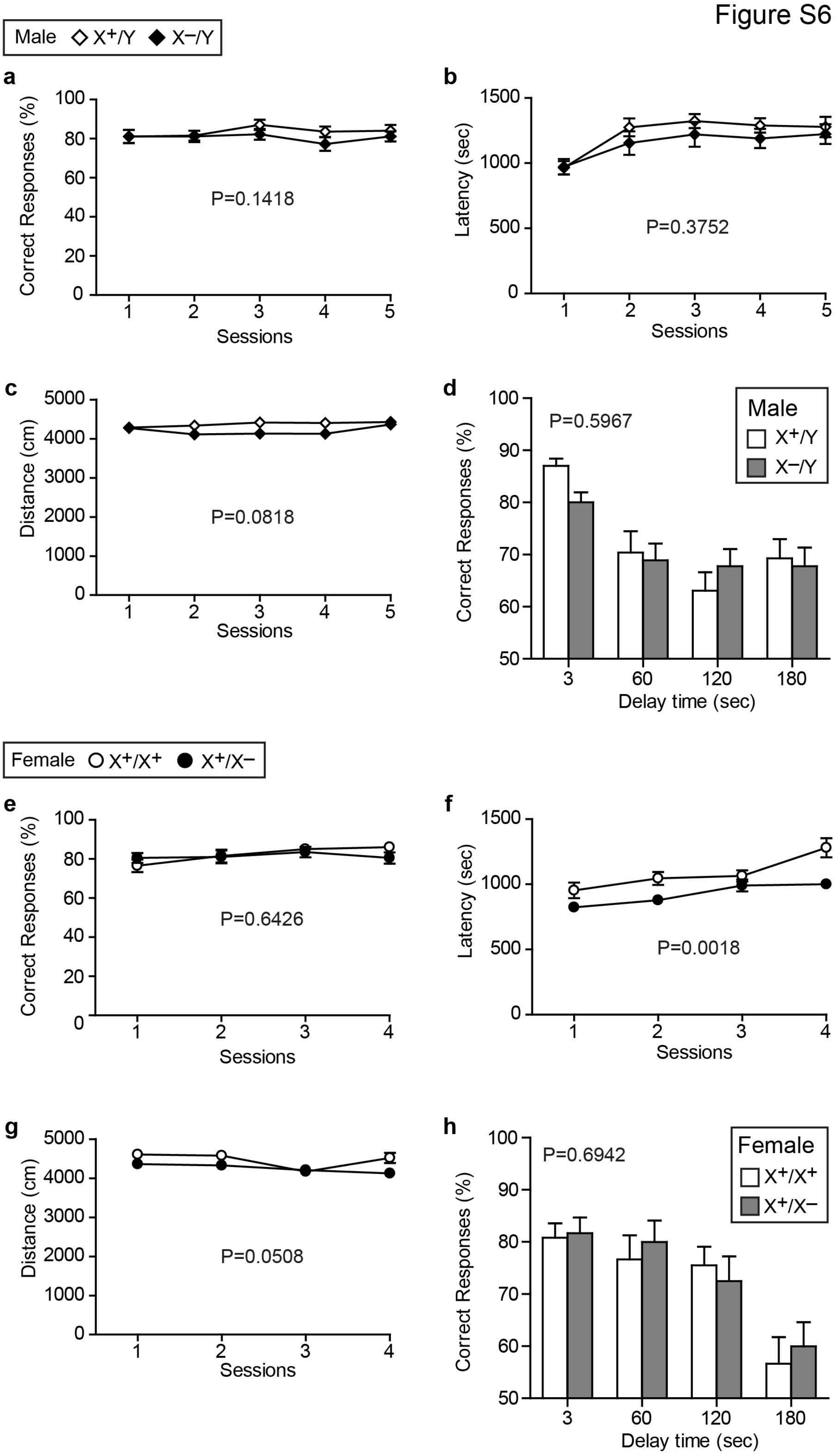

Male ◇ X+/Y ◆ X−/Y
a
Correct Responses (%)
Sessions
P=0.1418
b
Latency (sec)
Sessions
P=0.3752
c
Distance (cm)
Sessions
P=0.0818
d
Correct Responses (%)
Delay time (sec)
P=0.5967
Male
X+/Y
X−/Y
Female ○ X+/X+ ● X+/X−
e
Correct Responses (%)
Sessions
P=0.6426
f
Latency (sec)
Sessions
P=0.0018
g
Distance (cm)
Sessions
P=0.0508
h
Correct Responses (%)
Delay time (sec)
P=0.6942
Female
X+/X+
X+/X−

Supplementary Fig. S6 T-maze test.

(**a-d**) Test with male mice. (**e-h**) Test with female mice. Correct responses (**a, e**), latency (**b, f**), and distance traveled (**c, g**) were measured. (**d, h**) Delayed alternation test with male (**d**) and female (**h**) by 3, 60, 120 and 180 seconds before free choice run in the maze. There was no significant difference between wild-type and *Pcdh19* mutants in males or females.

Figure S7

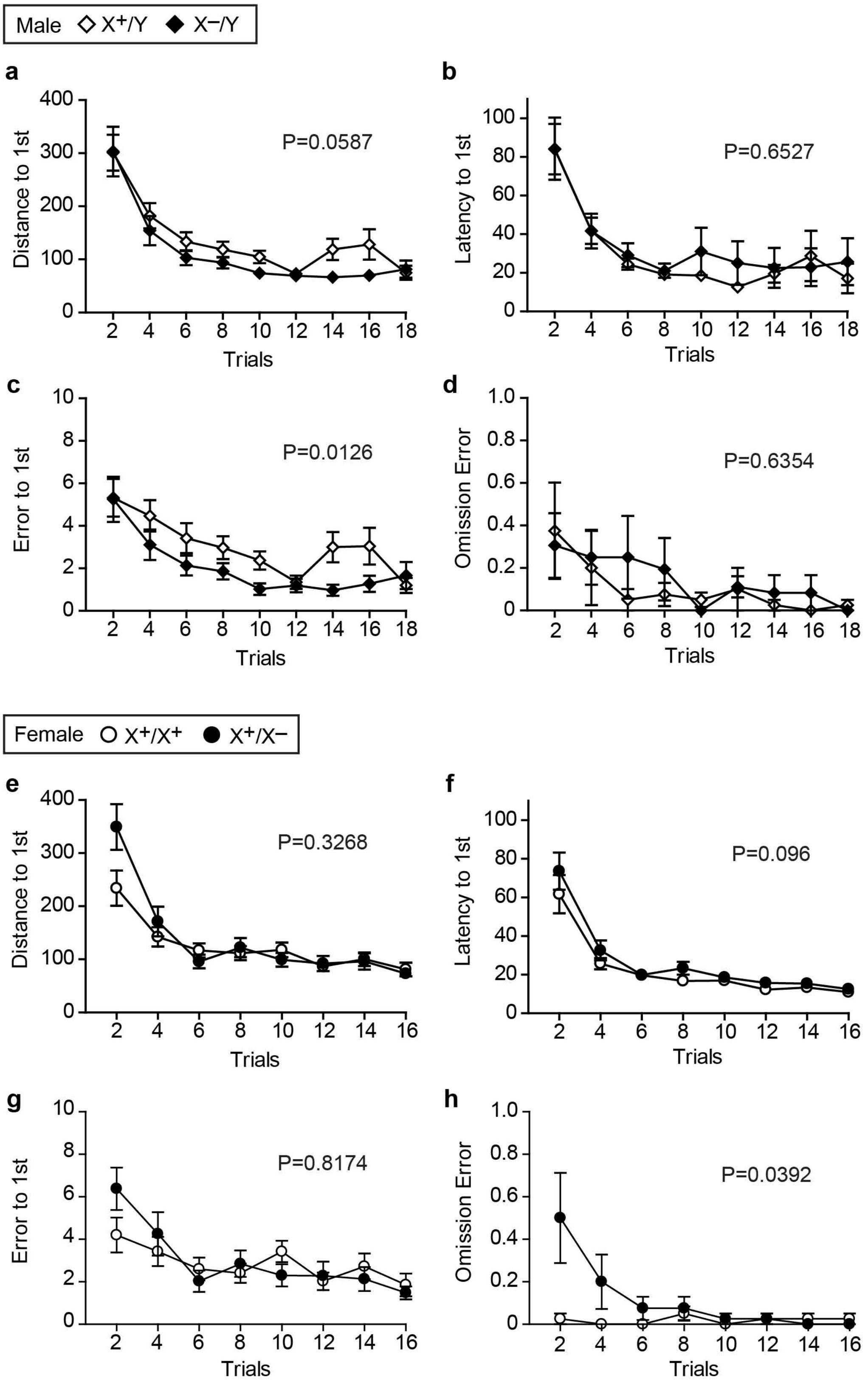
Male ◇ X^+/Y ◆ X^-/Y
a
Distance to 1st
P=0.0587
Trials
b
Latency to 1st
P=0.6527
Trials
c
Error to 1st
P=0.0126
Trials
d
Omission Error
P=0.6354
Trials
Female ○ X^+/X^+ ● X^+/X^-
e
Distance to 1st
P=0.3268
Trials
f
Latency to 1st
P=0.096
Trials
g
Error to 1st
P=0.8174
Trials
h
Omission Error
P=0.0392
Trials

Supplementary Fig. S7 Barnes test: training

(a-d) Test with male mice. **(e-h)** Test with female mice. The distance (**a, e**), latency (**b, f**) and the number of search errors made (**c, g**) to reach the target hole and also the number of omission errors (**d, h**) were measured. These parameters were gradually decreased during training over 18 days for males and 16 days for females. Although there were significant differences in the number of search errors in males (**c**) and the number of omission errors in females (**h**), these parameters finally reached the same level between wild-type and *Pcdh19* mutants.

Figure S8

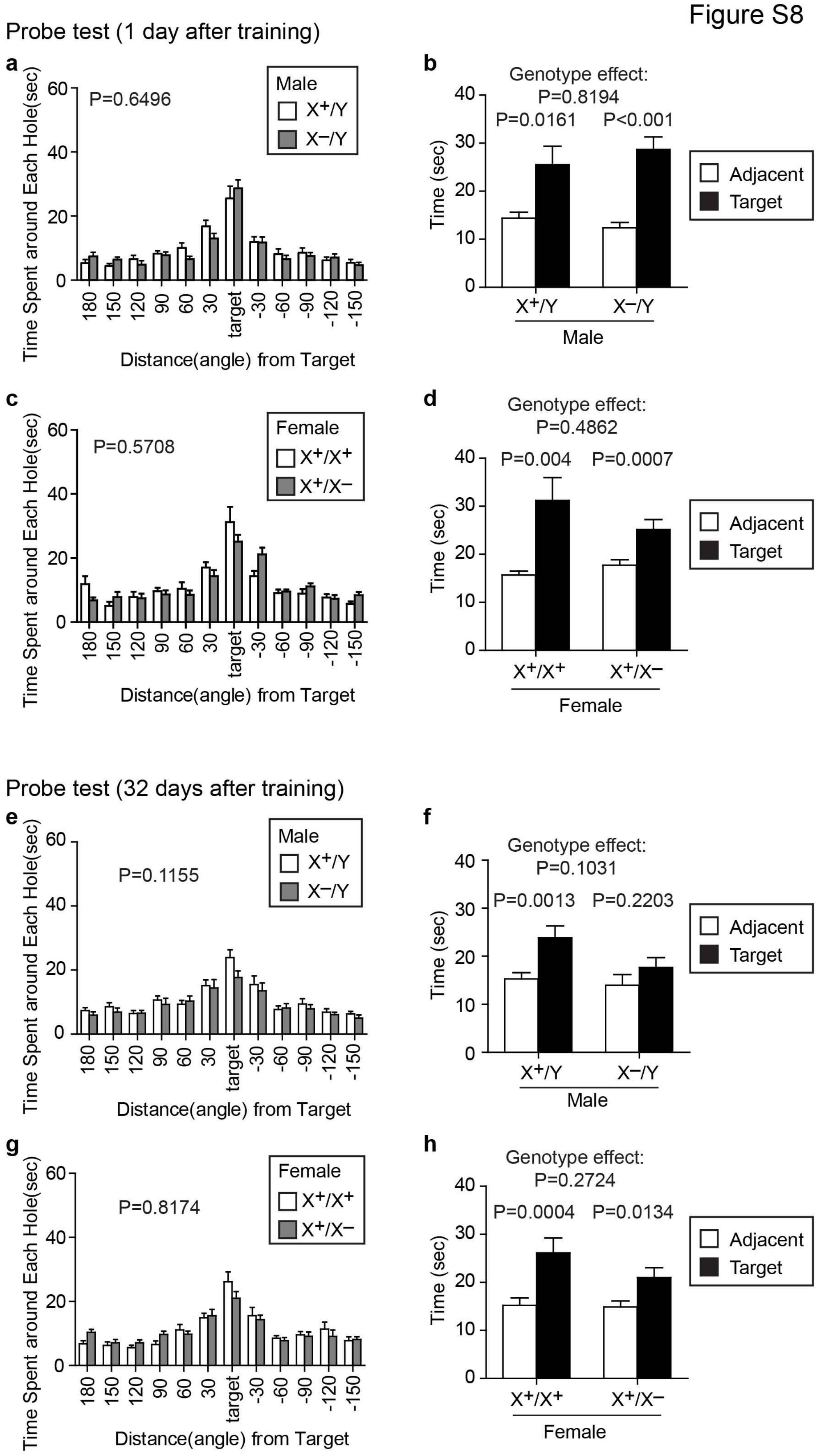
Probe test (1 day after training)
a
P=0.6496
Male
X+/Y
X−/Y
Time Spent around Each Hole(sec)
Distance(angle) from Target
180 150 120 90 60 30 target -30 -60 -90 -120 -150
b
Genotype effect:
P=0.8194
P=0.0161
P<0.001
Time (sec)
Adjacent
Target
X+/Y
X−/Y
Male
c
P=0.5708
Female
X+/X+
X+/X−
Time Spent around Each Hole(sec)
Distance(angle) from Target
d
Genotype effect:
P=0.4862
P=0.004
P=0.0007
Time (sec)
Adjacent
Target
X+/X+
X+/X−
Female
Probe test (32 days after training)
e
P=0.1155
Male
X+/Y
X−/Y
Time Spent around Each Hole(sec)
Distance(angle) from Target
f
Genotype effect:
P=0.1031
P=0.0013
P=0.2203
Time (sec)
Adjacent
Target
X+/Y
X−/Y
Male
g
P=0.8174
Female
X+/X+
X+/X−
Time Spent around Each Hole(sec)
Distance(angle) from Target
h
Genotype effect:
P=0.2724
P=0.0004
P=0.0134
Time (sec)
Adjacent
Target
X+/X+
X+/X−
Female

Supplementary Fig. S8 Barnes test: probe tests after training.

(**a-d**) Test with male (**a, b**) and female **(c, d)** mice one day after the last training. (**a**, **c**) Time spent around each hole was assayed. (**b**, **d**) The time spent around the target and the average of times spend around the adjacent holes (target ± 30°) were compared. There were no significant differences between wild-type and *Pcdh19* mutants in males or females. **(e-h)** Test with male (**e, f**) **and** female **(g, h)** mice 32 days after the last training. Parameters used were the same as those in **a**-**d**. There were no significant differences between wild-type and *Pcdh19* mutants in males or females.
